# Access to Essential Services: A Critical Factor for Predicting Recovery and Quantifying Resilience

**DOI:** 10.1111/risa.70323

**Published:** 2026-08-15

**Authors:** Tessa Swanson, Seth Guikema, Benjamin Nelson‐Mercer, Jeremy Bricker

**Affiliations:** ^1^ Industrial and Operations Engineering University of Michigan Ann Arbor Michigan USA; ^2^ Civil and Environmental Engineering University of Michigan Ann Arbor Michigan USA; ^3^ Civil Engineering and Geosciences Delft University of Technology Delft Netherlands

**Keywords:** access, disaster recovery, location‐based services data, machine learning

## Abstract

Essential services are necessary for individuals to recover from and adapt to disruptive events, yet the relationship between access to such services and recovery trajectory is poorly understood. Data on household recovery times are typically only available through surveys, which are limited in scale and scope. Further, the availability of all open and accessible essential services facilities in a region may not be recorded anywhere. Location‐based services (LBS) data from cell phones offers new opportunities for estimating this relationship. Using LBS data, we approximate facility availability and household recovery times following Hurricane Irma in Southwest Florida in 2017 and then statistically model the importance of access for recovery incorporating social vulnerability, local storm parameters, and infrastructure outage variables. We show that power, cell service, and school outages rank highest in importance, followed by measures of access to essential services. These results underscore the importance of including access metrics for predicting community recovery and evaluating resilience as well as the need for planning and policies that improve access to essential services both in times of stability and disruption.

## 1 Introduction

According to the United Nations Office for Disaster Risk Reduction's 2022 Global Assessment Report, “risk creation is outstripping risk reduction” (United Nations Office for Disaster Response 2022). The frequency and impact of disasters are increasing annually along with globalization, climate change, aging infrastructure, economic inequality, and coastal population growth (United Nations Office for Disaster Response 2022; Boustan et al. 2020; Changnon et al. 2000). Geographic, social, environmental, and economic characteristics of a household or community may exacerbate the risks associated with natural and man‐made hazards, with the most vulnerable communities presenting greater challenges throughout all phases of the disaster cycle: mitigation, preparation, response, and recovery (Boustan et al. 2020; Changnon et al. 2000; Green et al. 2007; Mitsova et al. 2018; Lee et al. 2022; Yabe et al. 2020; Sanders et al. 2023; Hendricks and Van Zandt 2021; Gardoni and Murphy 2020; Murphy and Gardoni 2006; Godschalk 2003; Bolin and Stanford 1998; Finucane et al. 2020; Turner et al. 2003; Karakoc et al. 2020; Singh et al. 2014; Hong et al. 2021). For a community facing a hazard, inequities carried over from pre‐hazard conditions may compound with those introduced by the hazard itself as well as recovery efforts that may reinforce previous inequities or introduce new ones through maladaptations (Murphy and Gardoni 2006; Godschalk 2003; Finucane et al. 2020; Rachunok and Nateghi 2021; Logan et al. 2021; Gunda et al. 2023).

Resiliency planning offers the opportunity to transform this paradigm. Resilience represents the capacity of a system to absorb changes and return to a previous state of progress or adapt to a new normal (Finucane et al. 2020; Rachunok and Nateghi 2021; Gunda et al. 2023; Aven 2011; Elmqvist et al. 2019; Meerow et al. 2016; Park et al. 2013; Koliou et al. 2020). Community resilience includes infrastructure, people, cultural facilities, and anything else that enables a system and all within it to thrive (Godschalk 2003; Karakoc et al. 2020; Gunda et al. 2023; Koliou et al. 2020; Logan and Guikema 2020). Achieving community resilience must integrate technical and social approaches to fully evaluate and mitigate risk across community members, infrastructure, and institutions (Gardoni and Murphy 2020; Murphy and Gardoni 2006; Allenby and Fink 2005). Frameworks for evaluating resilience rely on quantifying the disruption and recovery of a system, typically to its prior state (Frazier et al. 2014; Henry and Emmanuel Ramirez‐Marquez 2012; O'Hare et al. 2016; Hosseini et al. 2016). More recently, understanding resilience has come to include capturing mitigative and adaptive transformations prior to or following a disaster to avoid the “resilience trap” of returning to the inequality and vulnerability that were inherent to the original state (Rachunok and Nateghi 2021; Howell and Elliott 2018; O'Hare et al. 2016; Anderson et al. 2018; Sovacool 2017).

The qualities that define a community are inherently specific and hazards are unpredictably unique, complicating efforts to quantify and bolster resilience at localized levels. Literature on quantifying resilience typically falls into two distinct approaches: evaluating community capacity versus infrastructure functionality. Understanding resilience based on community capacity relies on indicators representing qualities that enable a community to mitigate, prepare for, respond to, recover from, and adapt following a disruption (Koliou et al. 2020; Cutter 2016). Such qualities include generalizing the population, economic assets, housing infrastructure, governance, and environmental characteristics of a community, as in the Social Vulnerability Index (SoVI) and Baseline Resilience Indicators for Communities (BRIC) (Cutter et al. 2014; Cutter 2016; Cutter et al. 2003). Though these indices are widely used for evaluating the capacity and resilience of communities, including for the US Federal Emergency Management Agency's National Risk Index (Cutter 2016; Cutter et al. 2014; FEMA n.d.; Spielman et al. 2020; CDC/ATSDR Social Vulnerability Index 2023), they have been criticized for internal and theoretical inconsistencies (Frazier et al. 2013; Tate 2012; Spielman et al. 2020; Rufat et al. 2019; Birkmann 2007). These measures are not event or hazard specific, lack temporal components, and assume that each of the included metrics equally contribute to the vulnerability of every community. Community vulnerability is place‐ and hazard‐specific, but these index values do not capture important contextual differences (Bakkensen et al. 2017; Spielman et al. 2020; Birkmann 2007; Fekete 2012; Beccari 2016; Goodman et al. 2021; Frazier et al. 2013). Furthermore, these index values are not particularly actionable, as they reflect relatively stationary demographic, infrastructure, and environmental characteristics of a community (Frazier et al. 2013; Spielman et al. 2020; Goodman et al. 2021). For these reasons, indicators like SoVI and BRIC often fall short in actually helping predict or mitigate the impacts of disaster outcomes (Bakkensen et al. 2017; Rufat et al. 2019; Spielman et al. 2020; Frazier et al. 2013).

On the other hand, infrastructure resilience is often modeled in terms of the built environment, specifying individual buildings or systems such as power networks that may lose functionality during a disruption (Henry and Emmanuel Ramirez‐Marquez 2012; Hosseini et al. 2016; Bruneau et al. 2003; Ganin et al. 2017; McAllister Therese 2016; Tariverdi et al. 2023). While these models have a physical basis for evaluating resilience, they do not capture the social consequences that impact a community when function is lost nor do they capture measures reflecting capacity to respond and adapt. There is a need for multidisciplinary approaches to quantify resilience in terms of a community's capacity to absorb, adapt, and recover from disruptions while capturing the inter‐dependencies across infrastructure, social, and economic system recovery and transformation.

### 1.1 Access to Essential Services

Access to essential services offers an approach for integrating both community vulnerability and engineering infrastructure–based approaches for evaluating community resilience (Logan and Guikema 2020; Tariverdi et al. 2023). Access to essential services, including water, power, healthcare, education, food, and cultural amenities are necessary for individuals to recover and adapt to disruptive events (Koliou et al. 2020; Logan and Guikema 2020; Cutter 2016; Frazier et al. 2014; United Nations Educational, Scientific and Cultural Organization and World Bank 2018; Loos et al. 2023; Tariverdi et al. 2023). Access to essential services is not the same across all members of a community even outside of hazard periods (Kullgren et al. 2012; Maharjan et al. 2022; Martens 2012; Widener 2018; Williams et al. 2020; Bills and Walker 2017). High mobility and access are linked to socioeconomic development including higher income and education rates as well as lower deprivation and unemployment rates (Pappalardo et al. 2015; Maharjan et al. 2022). When buildings, infrastructure, and transportation networks are damaged by a disaster, access is disrupted, whether through facility closures, transportation network barriers, or lack of adequate supplies. As a result, access measures can reflect locally specific inequalities in the ability of community members to acquire necessary resources and services both before and during the disaster, capturing the realized impacts of social vulnerability that shape recovery and are therefore fundamental to understanding and quantifying resilience.

Many studies have contributed to quantifying access, infrastructure criticality, and service essentiality (Tariverdi et al. 2023; Williams et al. 2020; Eckert and Shetty 2011; Swanson and Guikema 2023; Logan et al. 2019; Widener 2018; Fan et al. 2022). However, there is a gap in understanding how access, both preceding and following a disruptive event, influences household recovery time, particularly based on historical data from an actual disaster. Identifying the multiple dimensions of access across all these services is complex in itself (Kullgren et al. 2012; Levinson and Wu 2020; Saurman 2016; Penchansky and Thomas 1981), as well as requiring the collection of relevant data across a swath of service providers across the time period of the disruptive event. This is further complicated by the need to understand which services should be considered essential at what stages of recovery, relative to the timeline of the disaster and other recovery efforts at a fine resolution, such as individual households. Data on which facilities and transportation connections remained available during a disruptive event are typically unavailable unless collected from individual facilities and service providers in real time, and methods to obtain that data do not scale well to capture the broad geographic regions necessary to evaluate varying levels of community access and recovery periods while controlling for other disaster‐specific variables (Swanson and Guikema 2023). Thus, this relationship between access and recovery is not easily quantified, particularly beyond very aggregated populations (Logan et al. 2019;2023; Cutter et al. 2014; Tariverdi et al. 2023; Fan et al. 2022). Addressing this gap requires acknowledging the criticality of access measures in the first place to motivate including access‐metrics in resiliency planning and response efforts as well as collecting appropriate data to quantify household access to essential services before, during, and after hazardous events. This paper aims to bridge this gap by providing empirical evidence of the importance of access metrics in predicting household recovery time and illustrating the relevance of access for future efforts to quantify resilience.

### 1.2 Location‐Based Services (LBS) Data

LBS data from cell phones offer new opportunities to capture the relationship between behavioral responses to disruptions and access to essential services facilities in the periods surrounding a disruptive event. Historically, understanding behavioral response to disasters has relied on surveys (Calabrese et al. 2013; Wang et al. 2012). But capturing diverse experiences of a disaster over a time horizon including all preceding and following behavioral patterns requires an extensive sample size or focus on a very specific community as well as either the foresight to initiate a survey months in advance or reliance on retroactive reporting, which is subject to recall bias. LBS data come from opportunistically collected user locations from cell phone interactions with a cellular network or WiFi, thus providing a mobility profile of all included users with a sample size several orders of magnitude larger than can be feasibly obtained by surveys. LBS data have been demonstrated to be sufficiently representative to capture diverse users across a large geography, socioeconomic distribution, and path of a disruptive event despite not including any user demographic data (Cinnamon et al. 2016; Yabe, Sekimoto, et al. 2019; Chen et al. 2016; Chen and Rohla 2018; Washington et al. 2023; Marzuoli and Liu 2019; Yin et al. 2020; Long et al. 2020; Yabe et al. 2022). Many studies have successfully observed behavioral responses to disasters from LBS data, but none have yet connected those responses to corresponding changes in access to essential services (Washington et al. 2023; Marzuoli and Liu 2018; Lee et al. 2022; Jones et al. 2018; Yabe, Ukkusuri, et al. 2019; Yabe, Sekimoto, et al. 2019; Yabe et al. 2020;2022; Podesta et al. 2021; Cinnamon et al. 2016; Yin et al. 2020; Mackrell et al. 2013; Dobra et al. 2015; Wilson et al. 2016; Hong et al. 2021). Such a vast number of observations is conducive to statistical modeling even with a large number of features, including variables representing access to essential services, enabling new possibilities of inferential and predictive modeling to understand the underlying behavioral patterns reflected in the data.

### 1.3 Contribution

In this paper, we statistically model variables influencing household and workplace recovery times utilizing LBS data from Veraset, LLC spanning August 1 to October 3, 2017, in Collier, Hendry, Lee, and Monroe counties in Southwestern Florida. On September 10, 2017, Hurricane Irma made landfall in Southwest Florida as a Category 4 Hurricane, devastating the Keys in particular but causing widespread power outages and facility closures throughout the state (Mitsova Diana et al. 2021). Power restoration took up to 3 weeks in some communities and schools in Monroe County closed for 3 weeks, from September 6 until September 27 (Gurney and Miami 2017). We identify the locations of essential services facilities from OpenStreetMap, including supermarkets, libraries, convenience stores, gas stations, pharmacies, clinics, hospitals, and home improvement stores (OpenStreetMap contributors 2023).

We identify user home and work locations based on a method described in Chen et al. (2014) and Washington et al. (2023) and apply a Bayesian network–based anomaly detection algorithm to estimate when users experienced recovery from their home and work visit patterns, where recovery periods are defined based on methods developed by Swanson and Guikema (2026) and further delineated for this application in Section 4.1. We then apply an LBS‐based functional closures algorithm to identify when the facilities were closed or otherwise inaccessible during the period surrounding Hurricane Irma based on Swanson and Guikema (2023), described further in Section 4.2. We then calculate the travel times between each home and work location and the previously identified essential services facilities using OpenTripPlanner (Malcolm Morgan et al. 2019), described in Section 4.3. From these results, we determine each user's travel time from their home and workplace to all open essential services facilities within 30 miles for each day in the month of September 2017 to capture changing potential access (defined as the travel time required to access the nearest accessible service of a given service category) to these essential services facilities. Individuals may not always visit the nearest facility, so for each day in the study period we evaluate both travel time to the nearest facility as well as the total number of available facilities within threshold travel times (ranging from 10 to 60 min), capturing a broader representation of accessibility. Both of these metrics serve as a general proxy for potential access by capturing the range of available options, recognizing that in emergency scenarios individuals may rely on any available facility rather than single preferred locations. By covering the evolving availability of services over the study period, these measures move beyond simple geographic accessibility toward potential realized access, reflecting the set of options that remain practically available to users under prevailing network conditions.

Finally, as detailed in Section 4.4, we build out a database to include these daily metrics representing potential access and recovery status as well as socioeconomic and SoVI data from user's home and work census tracts, storm variables capturing wind and storm surge, and county‐level power and cellular network outages. From this data set we develop random forest models to estimate the impact of all of these variables on recovery status and assess the importance of including of access to essential facilities metrics in predicting recovery. Using these LBS data methods together for the first time, we are able to present the novel quantification of the importance of access to essential services relative to other storm, infrastructure, and social vulnerability measures. Based on these results, we argue for quantifying resilience as a function of access‐based measures, motivating the inclusion of access measures to augment existing vulnerability indices across all stages of resiliency planning and hazard response.

## 2 Results

### 2.1 Changes in Access

**FIGURE 1:**
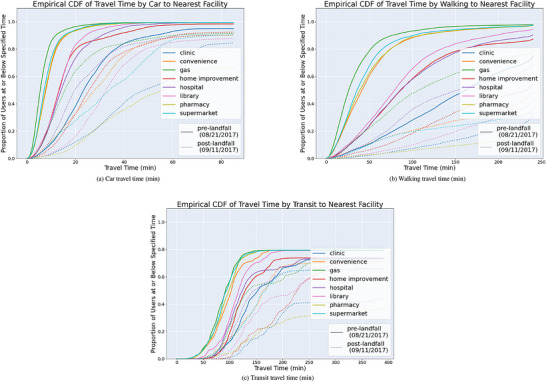
Empirical cumulative distributions of travel time by mode.

In Figure 1, we show empirical cumulative distribution plots (ECDF) of how travel time to the nearest facility—used as a proxy for potential access—for each type of essential service compares between a typical day prior to landfall (August 21, 2017) and 1 day post‐landfall (September 11, 2017) by each mode.

Figure 1 shows that access in terms of travel time and availability drastically decreases the day following Irma's landfall. Even before Irma, transit travel times for most individuals were very high, with only about 25% of home and workplaces within 60 min of a supermarket, gas station, convenience store, or pharmacy and fewer than 10% of home and workplaces within 60 min of a library. Furthermore, transit access was determined by allowing up to a mile of total walk time, resulting in fewer than 80% of users having any transit‐based route to supermarkets, gas stations, pharmacies, convenience stores, and libraries, with an even smaller share of users for other facility types. Notably, the increase in transit travel time following Irma's landfall does not account for transit services being closed. Walking to essential services is also notably inaccessible even before landfall, as walking more than 60 min is not accessible for most individuals for most trips. About 50% of users have a gas station, supermarket, pharmacy, or convenience store within 30 min, but libraries, hospitals, and clinics are much further.

**FIGURE 2:**
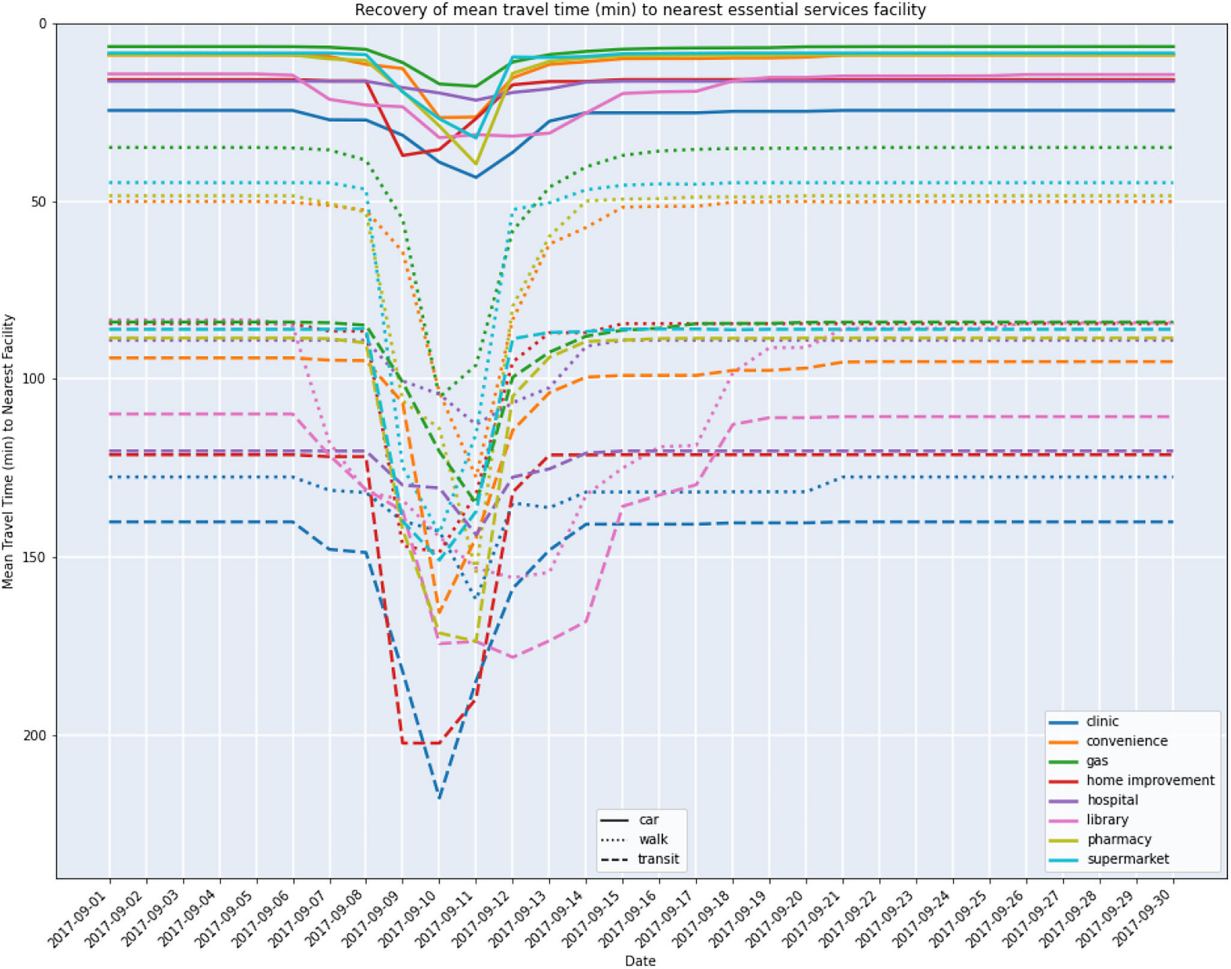
Travel time recovery curves by facility type and travel mode.

We show the change over time of the mean minimum travel time (again as one proxy for general access) to each facility type by each mode type in Figure 2.

Figure 2 shows how access to each facility type recovers in the days following Hurricane Irma's landfall, with gas stations showing the least decrease in access and quickest recovery, while libraries had relatively long recovery periods. Walking access had the greatest change between pre‐ and post‐landfall travel times, tripling for most facility types. Recovery curves broken down by facility type and travel mode can show decision‐makers differences in how access is being restored, which may be useful for determining priorities for recovering individual facilities and connections in the transportation network.

**FIGURE 3:**
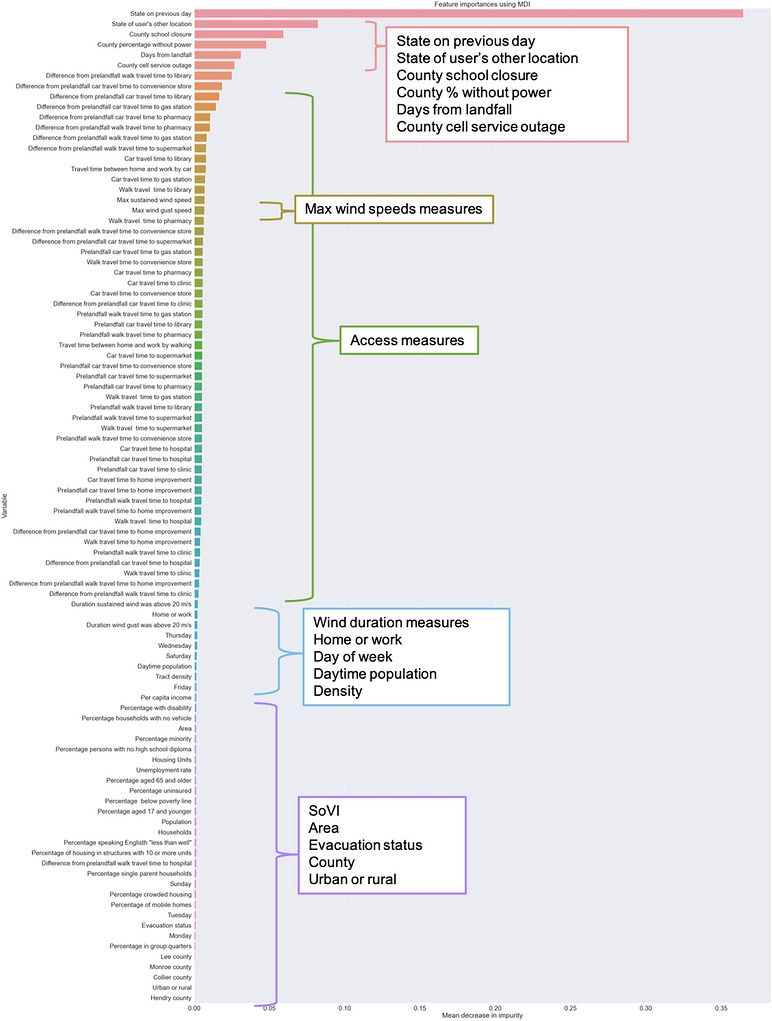
Importance for random forest model including Monroe, Collier, Lee, and Hendry counties.

We developed prediction models initially estimating home and work recovery periods for 7639 users across Monroe, Collier, Lee, and Hendry counties with 102 variables. Additionally, we developed and ran a storm surge model for Monroe and Collier counties, described in Appendix A, so also evaluated models that included five storm surge variables for the 2905 users in Monroe and Collier only because only these two counties experienced appreciable storm surge. We conducted a cross‐validation process for variable selection and to determine a class weighting method. Detailed cross‐validation model results are described in Appendix B. Cross‐validation results were consistently highly accurate across all tested scenarios, with average out‐of‐sample test set accuracy ranging from 94.2% to 94.7%. With these results in mind, we trained a model using the users from all four counties with balanced subsampling and with 102 total variables representing the days since landfall, whether the location was home or work, the user's state on the previous day, the user's state at other location, county‐level power outages, county‐level cell network outages, county‐level school closures, zone evacuation status, 56 driving and walking access measures, 19 social vulnerability measures, population, area, density, urban or rural classification, four wind speed variables, county, and day of week. The importance ranking of this model is plotted in Figure 3. The corresponding labels on the plot generalize the themes of the individual variables within each bracket.

**FIGURE C.1:**
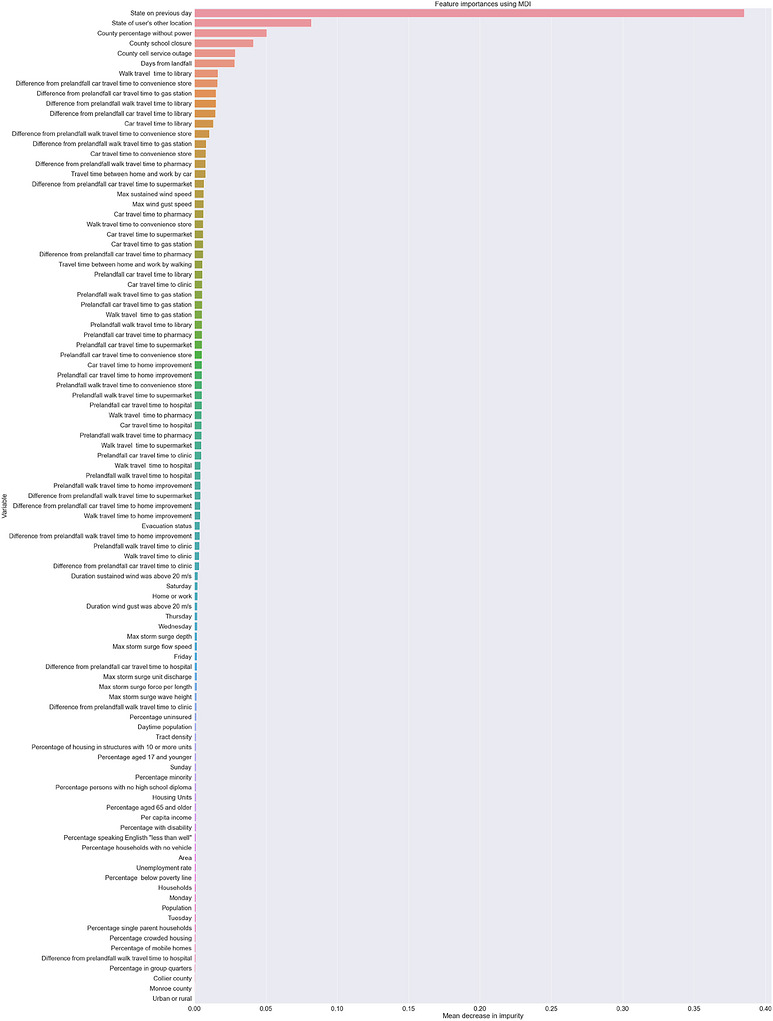
Importance for random forest model including only Monroe and Collier counties and storm surge variables.

The importance rankings of previous state and the user's other location state stand out, followed by school closures, percentage of county without power, days since landfall, and cell network outages. These variables are then followed by several access measures denoting the change in access from pre‐event travel times. Notably, multiple measures of access to libraries were ranked highly and libraries were among the last of facilities to open based on Figure 1. Libraries are also the only public services among the buildings we included. Wind speed variables are ranked 19 and 20 followed by many more access variables, then variables representing day of week, density, SoVI values, and county designation. We also developed a model for only Monroe and Collier counties to include the storm surge variables, though all of the storm surge variables ranked relatively low in importance, shared in Appendix Figure C.1. While wind and storm surge variables were both included to control for damage, storm surge is highly localized along the coast whereas wind speed and duration impacts are more widespread across coastal and inland geographies. Furthermore, wind can contribute to downed trees and power lines that have system‐level impacts that would impact recovery time.

To evaluate if different variables impacted home and work recovery differently, we generated additional models, with the same architecture, separately for home and workplaces, shared in Appendix D. The workplaces model ranks power outages higher than school closures and days‐since‐landfall shifts up above cell outages, but otherwise both the home and workplaces models showed importance rankings consistent with Figure 3. We also developed separate models for users with homes in urban versus rural areas though only 496 users, about 6.5% of the data, have homes classified as rural based on Florida Department of Transportation definitions of urban boundaries (FDOT Open Data Hub 2024). As expected, the urban results resemble the full model, while the rural results show higher importance for school closures and slightly higher ranking of difference in travel time to the closest open supermarket. These results are also shared in Appendix E. Finally, as the importance rankings are dominated by the previous state and the user's other location state variables, we test the sensitivity to excluding each of these variables from the previously described model with all four counties. The importance values generated from excluding previous state and other location state features are shown in Appendix F. When excluding the previous state variable, the accuracy decreases to approximately 91% and the ROC‐AUC value decreases by 0.019. Still, the ranking order shifts relatively little other than wind gaining slightly more importance. Notably, access measures are still positioned much higher than the SoVI measures. When the other state variable is excluded, the ROC‐AUC value decreases by 0.037 and again the ranking metrics stay relatively similar other than the wind variables shifting up in importance ranking. These results instill confidence in the predictive contributions of all other included features.

**FIGURE 4:**
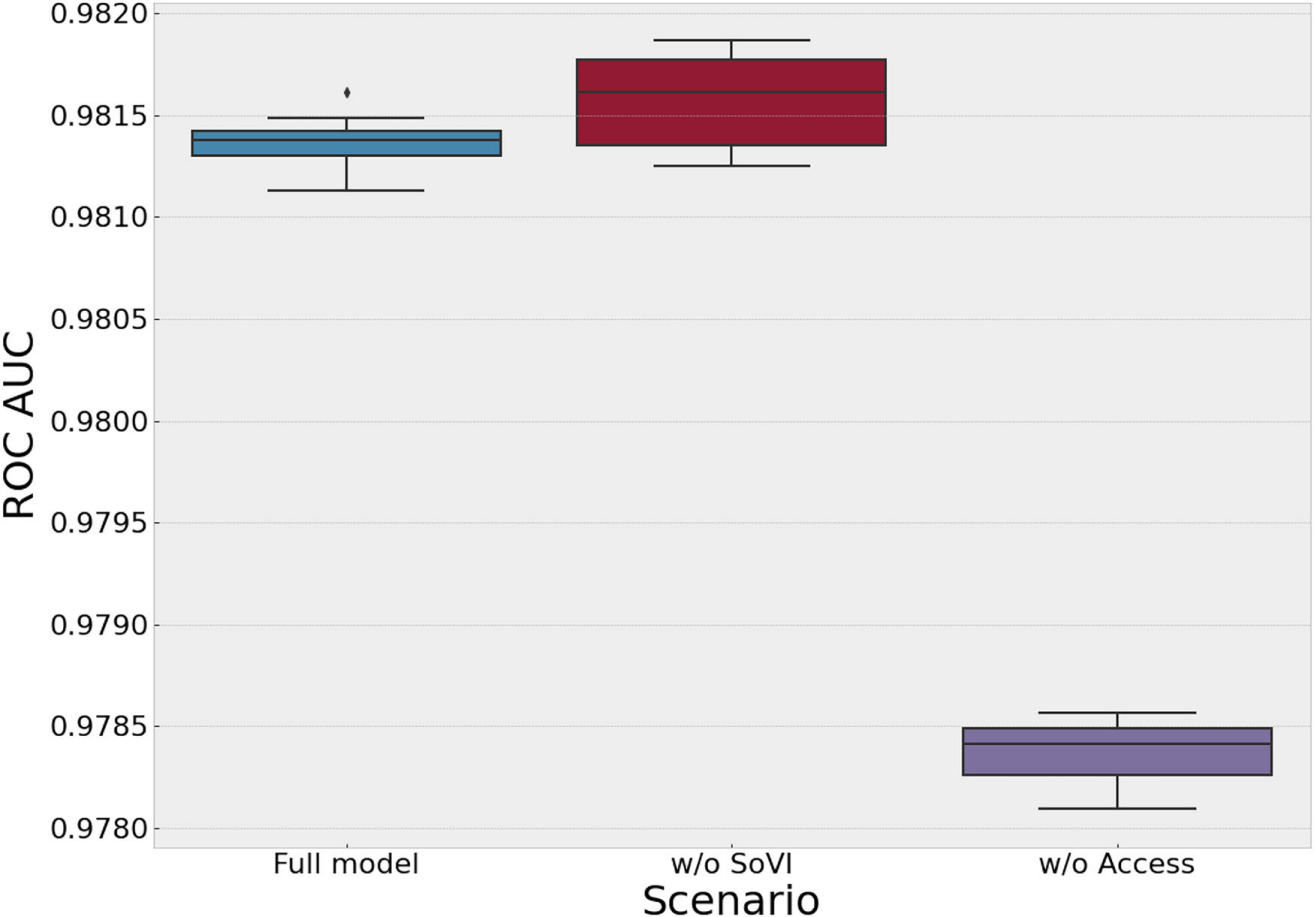
Comparison of ROC‐AUC performance for models with and without SoVI and access variables.

We conducted statistical comparison of the full model versus a version without SoVI variables and another without access variables. A two‐sample *t*‐test comparing the ROC‐AUC values for 10 cross‐validation sets for each model revealed a statistically significant, though slight, increase in performance when SoVI variables were excluded (*p*‐value = 0.021). When access variables were excluded, there was a much larger and statistically significant decrease in performance (*p*‐value < 0.001). These results are shown in Figure 4 below. We also share comparison of F1 performance, which shows a similar pattern, in Appendix G.

## 3 Discussion

These results indicate the value of including access variables over more traditional social vulnerability measures for predicting household‐ and workplace‐ level recovery times. These results do not discount the impact of social vulnerability on hazard experience, but indicate that static and non–hazard‐specific SoVI values do not help predict recovery nearly as much as measures of access to essential services. The relative contributions of any social vulnerability characteristic to resilience and recovery may vary drastically by location and by hazard (Spielman et al. 2020). The household vulnerability in one community may not even be consistent with other communities in the same region (Spielman et al. 2020; Birkmann 2007; Frazier et al. 2013), and so these metrics are not necessarily useful for quantitatively evaluating resilience and recovery. This is even more true for the aggregate theme variables (summary rankings of the four vulnerability themes) and flag variables (counts of indicators for which a tract falls in the top 10% of vulnerability) which are currently used to rank the relative vulnerability of census tracts (CDC/ATSDR Social Vulnerability Index 2023). Meanwhile, access variables capture the systemic differences in access that may be specific to a given community. For example, racial disparities in food access are prevalent in many communities, but race is not consistently a good predictor of hindered food access everywhere (Widener et al. 2017; Eckert and Shetty 2011). Access measures capture the realized impacts of social vulnerability specific to a location and provide actionable steps for decision‐makers during all phases of a hazard. During the preparation phase, knowing the areas within a community with already low access or with high likelihood of diminished access can help with prioritizing the distribution of supplies prior to an event. In the recovery phase, access metrics can guide decision‐making around the restoration of infrastructure and essential services facilities, as well as guide where to create temporary locations to provide supplies for immediate needs (Svirsko et al. 2022; Fan et al. 2022). Over the long term, incorporating access into resiliency planning can also promote transformation, or a systemic change of an existing system, through mitigative and adaptive measures to avoid resiliency traps that often reinstitute inequities when communities strive to recover to an already un‐resilient baseline (Boustan et al. 2020; Rachunok and Nateghi 2021; Elmqvist et al. 2019; Logan and Guikema 2020; Sovacool 2017). Such transformative mitigation and adaptation may involve long‐term planning around equitable access to essential services through prioritizing facility openings and transportation options based on how they contribute to improved community resilience. Thus, understanding the relationship between access and community resilience could contribute to motivating improvement in access at every stage of the hazard cycle for the households and communities who need it most.

### 3.1 Limitations and Opportunities

This method is limited by the available data and tools. Given LBS data do not include demographic information, there is no way to ensure representativeness of the studied population. In 2017, 82.8% of Florida households owned a smartphone (U.S. Census Bureau 2017). Washington et al. (2023) performed regression analysis on county‐level representativeness of the same 2017 LBS data that were used in this study covering the Southeast United States. They showed that inclusion of variables representing percent Asian, percent White, percent Hispanic, and percent over 75 years old marginally but statistically significantly improved model accuracy, indicating some bias in the data set that could be attributed to differences in sociodemographic variables, location, smartphone ownership, phone use patterns, or applications sharing location data. Evaluating representativeness is a critical step for any research utilizing LBS data, especially considering the intersection of population vulnerability with rates of smartphone ownership. In addition to potential bias in LBS data, the results presented in this paper also rely on open source data and software, including OpenStreetMap and OpenTripPlanner, which may be incomplete and suboptimal in some locations. OpenStreetMap is widely relied on in various research studies for street network and point‐of‐interest data (Yabe et al. 2022; Barrington‐Leigh and Millard‐Ball 2017; Wang and Taylor 2014; Yu et al. 2018; Logan and Guikema 2020; Logan et al. 2019; Toole et al. 2015; Mooney et al. 2021), but as an open source data set offers quality and completeness concerns, especially in rural areas (Hecht et al. 2013; Vargas‐Muñoz et al. 2019). Coverage and accuracy are improving over time, but coverage and accuracy of OpenStreetMap data should be checked and considered for evaluating future study areas.

The functional closures algorithm only serves as a proxy for what facilities were inaccessible and when; the true closure periods of those facilities and of transportation networks are not known. Furthermore, this model does not currently incorporate access to shelters or pop‐up suppliers that are known to provide emergency assistance in the days following a disrupted event. Restoration timelines of infrastructure networks varied highly within communities, as in Mitsova et al. (2018), but we only have access to power and cell network outages data estimated at the county level and so cannot speak to the more granular impacts of infrastructure damage and outages on recovery. Finally, the exclusion of transportation and transit network outages is a missing component in this study, particularly for estimating travel times as the shortest route to an open facility as estimated by OpenTripPlanner may not be available. The results of our model motivate efforts to enhance open data and software platforms to achieve greater precision and accuracy, as well as for infrastructure agencies and utilities to provide open access to ground truth local outage and closure data throughout the duration of their restoration efforts.

This research inspires opportunities for developing these ideas and models further. Workplaces could be classified further based on the land‐use of the location, especially in states or counties where land‐use is highly regulated. Other access measures could also be incorporated, as described by Saurman (2016) and Penchansky and Thomas (1981), like the availability and accessibility of supermarkets accepting SNAP and WIC as an additional measure of affordability. This work could be extended to capture the relationship between access to essential services and recovery for other geographic regions and hazardous events. Of special interest are communities with higher transit dependence and transit service levels, which would be expected to include other behavioral responses to disruption, as was the case with transit use and recovery following Hurricane Sandy (Kontou Eleftheria et al. 2017). Finally, future work with LBS data could capture realized access beyond just potential values of travel times and number of services within a time threshold, identifying the services users actually visit and utilize in the periods following a disruptive event (Shao and Luo 2026). With more data representing access to essential services and change in access over time, additional data sets and model configurations may be able to help answer more specific questions about what services are needed, when they are needed, and how access varies across different hazards and communities beyond Southwest Florida.

### 3.2 Implications for Policy and Research

Measuring resilience in terms of access offers new opportunities for integrating infrastructure and social capacities within a community system. FEMA already has GIS‐based tools for estimating hazard risk and impacts. The work presented in this paper motivates building similar tools, like urban digital twins, to include access measures for a more comprehensive and place‐specific portrait of hazard risk. With such tools in hand, planning and emergency response decision‐makers can build on traditional property loss assessments by incorporating access to essential resources, supporting a more holistic understanding of recovery.

The work described in this paper can also motivate local decision‐makers to evaluate access in their own communities with known context of the specific social vulnerabilities that may exacerbate risk. Quantifying resilience in terms of access presents opportunities for operationalization by local decision‐makers at every stage of a hazard, but requires transdisciplinary buy‐in and expertise for effective implementation. For the hazard mitigation stage, access to essential services, equity of access, and redundancies and robustness of access are all quantifiable and actionable metrics that can be evaluated to determine where additional essential services facilities or transportation connections may improve resilience (Fan et al. 2022; Svirsko et al. 2022). For preparation, knowing specific locations with already low access to certain resources or with high likelihood of decreased access can aid the prioritization of the distribution of provisions. During the response and recovery phases, anomaly detection applied to available data through methods like those presented in Swanson and Guikema (2023) and Swanson and Guikema (2026), may show which facilities and households are recovering and in need, and can provide a real‐time picture of changes in access to essential services over time. This work also motivates the need for additional data streams on access, as LBS data are helpful for retrospective analysis but may not be available to practitioners. With additional data sources reflecting access metrics, emergency responders can know where to place temporary supply stations, while decision‐makers can prioritize infrastructure and facility restoration to improve access where it could be most effective. Transformative sustainability planning can involve incorporating equitable access to essential services in long‐range planning to compare commercial facility location and transportation plans based on how they impact access and thus community resilience. With such knowledge at each hazard stage, decision‐makers can introduce community‐centered, place‐based interventions, and policies to promote more equitable and resilient communities and diminish the scale of disruptions. To get to that point, more work is needed to determine exactly which services are essential for who and at what time. Successful fulfillment of this work will require cooperation across social scientists, planners, policymakers, engineers, and data scientists to ensure quality and inclusive data and methodologies that account for the breadth and depth of human experiences to develop effective tools and policies that contribute to a more resilient future.

## 4 Methods

### 4.1 Estimating Household and Workplace Recovery

We determined the locations of homes and workplaces from LBS data using an algorithm developed by Washington et al. (2023). For each user, we identified points between 9 p.m. and 6 a.m. for determining homes and between 10 a.m. and 3 p.m. for determining workplaces, including only users who appeared at least 10 unique days of the 61 days in August and September 2017. We then overlaid 5e‐5 degrees latitude by 5e‐5 degrees longitude grid, creating clusters from all user points in neighboring grid cells (i.e., all points included in 1.5e‐4 by 1.5e‐4 degree overlapping grid cells). We select the cluster visited on the greatest number of unique days, and for users with multiple clusters at this point we select the cluster with the highest total duration spent (based on sequential appearances in the same cluster) during the selected time period (9 p.m. to 6 a.m. for home, 10 a.m. to 3 p.m. for work). When a user's workplace is within 0.0015 degrees (about 0.1 miles) of home, we select the second‐most visited location that still matches the prior criteria. We filter out any users with a horizontal accuracy less than 1 mile (about 0.1% of remaining users), users who do not appear at their home a week prior to landfall (42% of remaining users), and users who appear in the data set fewer than 80% of the days in August and September 2017 (19% of remaining users) to be sure we are only including users with enough daily data to identify visit patterns. In the end, we identify both home and work locations for 123,445 unique users. This is equivalent to about 0.5% of Florida's population in 2017, far exceeding the feasible sample size of a survey. We validated home locations against the Florida 2017–2021 American Community Survey (ACS) 5‐year population estimates (U.S. Census Bureau 2021) and workplaces against the Census Tract Planning Products (CTPP) program tabulation of worker flows published in 2017 (of State Highway and Officials 2017), both at the census tract level. Using simple linear regression to estimate ACS (or CTPP) tract density transformed to log scale from LBS home (or work) locations, tract density transformed to log scale yielded *R*‐squared values of 0.88 and 0.90 for home and work, respectively. With this result, we proceeded with our analysis confident that our identified home and work locations were sufficiently representative.

For each user, we then generated time series based on whether they appeared at home, work, somewhere else in their home county, or “other” for appearances somewhere else in Florida. We developed a Bayesian belief network (BBN) incorporating conditional probabilities of an appearance given day of week, whether the day was a weekend/holiday, and appearances on previous 3 days, as well as joint probabilities of home county and “other” locations to estimate the probability of an appearance at home (or work) on a given day. We used all users' August data to develop a prior probability distribution for every combination of nodes in the BBN. Then, for each user we update that prior distribution using the individual user's August data, weighing the prior to be the equivalent of one additional day. This step is taken in order to account for every possible combination of events, even if an individual user does not display all possible combinations of events in their own August data. We then applied the probability distribution to each user's September data to estimate the likelihood of each day's event (an appearance at home/work or not) given all nodes in the BBN. Any extremely low probability event (three standard deviations from users' observed August event's mean log probability) was identified as anomalous and we classified series of anomalous points as anomalous segments. We then designated recovery periods as the longest of those segments with a start date between September 6, 2017 (the first day of evacuation notices), and September 13, 2017, that ended at the beginning of three non‐anomalous days observed in a row. A binary was determined for each user for each day to indicate whether a user was within that recovery period (denoted as 1) or outside of it (denoted as 0) (Swanson and Guikema 2026).

### 4.2 Facility Closures

We first identify facilities that may have closed or otherwise became inaccessible. Lack of access may result from the facility declaring itself to be closed (e.g., due to direct damage, lack of employees, or loss of infrastructure services such as water or power), from disruption to the connecting transportation system, or from so few people accessing the facility because of nearby evacuation or stay‐at‐home orders. Using OpenStreetMap (OpenStreetMap contributors 2023) and the OSMnx Python package (Boeing 2017), we select all facilities in the state of Florida tagged with the following categories: supermarket, school, pharmacy/chemist, hospital, clinic, doityourself (e.g., home improvement and hardware stores), library, convenience, and gas. To include as many locations as possible, we downloaded all facilities represented by points, polygons, and multi‐polygons in the year 2023, which include 34% more locations within those categories as in 2017, the year of Irma's landfall. While OpenStreetMap data are open source and are not validated against another official data set for the study area, as of 2024, Florida has relatively high point‐of‐interest coverage, in terms of proportion of untagged buildings at 89%, the second highest in the United States (de Arruda et al. 2024). Still, to ensure we were being as comprehensive as possible given the limitations of this data set, we elected the most generalized tags that capture the aforementioned categories. For healthcare facilities, hospitals were identified using amenity tag “hospital” which denotes the inclusion of in‐patient care while clinics were identified based on the amenity tag “clinic” which generally represent outpatient medical centers that do not provide inpatient care and may include a range of healthcare providers, specialty practices, diagnostic centers, and urgent care facilities. As a result, the healthcare access measures used in this study capture potential access to broad categories of healthcare services rather than access to a specific primary care provider or medical specialty. We acknowledge that healthcare access is inherently multidimensional and may also depend on factors such as provider availability, insurance acceptance, and appointment scheduling, which are not captured by the travel time–based measures used here.

To generate a footprint for the facilities represented as points, we calculated the average footprint area of the polygons and multi‐polygons that were available. We calculated a radius for each category to apply a buffer around the associated points that would create a footprint equal to that category's average area. Note that if there is a bias in the OpenStreetMap data, like if smaller facilities are more likely to be recorded as points, then this may introduce a systematic error like overestimating the footprints of smaller facilities. Next, we applied an LBS‐based functional closure detecting algorithm (as described in Swanson and Guikema 2023) to these footprints to generate a time series of unique users appearing within the footprints on each day in August 1 to October 3, 2017. We filtered out any facilities that did not have 10 or more unique users on at least 19 days in the study period (i.e., we only included facilities had regular users at least 3 days per week prior to landfall). This filter removed 37% of convenience stores, 28% of coded libraries and clinics, and 25% of gas stations. As library locations are centrally published on county web pages, we wanted to ensure their inclusion and so manually added locations for seven libraries not included in the OpenStreetMap data. Through this filtering process, any facilities that were constructed after 2017 would not have enough detectable visit patterns, and so would be discarded through the algorithm. Any facilities that were repurposed after 2017 may be misrepresented in the locations data and so may be a source of error in subsequent analyses. Furthermore, this filtering may disproportionately exclude smaller or infrequently visited facilities, particularly in rural areas, potentially biasing estimates of access toward larger facilities. We acknowledge this as a limitation of this study.

To identify closure periods, we applied a pruned exact linear time (PELT) anomaly detection method from Killick and Eckley (2014a) available through the “changepoint” R package (Killick and Eckley 2014b) to each facility time series, excluding schools (given county‐level data on closure dates were available for public schools, we encoded school closure periods manually). PELT is a dynamic program that identifies changepoints within a time series based on a statistically significant change in the linear parameters of the series. For a Poisson distribution, like these time series counting user appearances, this means a statistically significant difference in both the mean and variance of neighboring segments. We applied the PELT algorithm and flagged all segments that contained September 10, 11, or 12. We determined a closure period as a flagged segment that had a mean less than both the prior and following segments and that started between September 3, 2017, and September 12, 2017. We interpreted significant decreases in the distribution and mean of facility visits as indicative of lack of access, which could result not only from explicit facility closure, but also from diminished access caused by disruptions to transportation systems or stay‐at‐home orders. This approach accounts for periods when facilities are effectively inaccessible, even if not officially closed, and helps to filter out noise by considering more than just instances with zero visits. Only 24 of the 626 non‐school facilities had no detected closure, zero of which were in Monroe County and only six of which were in Collier County. Of those six, four were hospitals that each showed an increase in visits in the immediate days surrounding landfall, indicating continued access to these facilities.

### 4.3 Travel Time

From every household and workplace, we found the travel time by car, walking, and transit to every facility within 30 miles using the open source routing software OpenTripPlanner (Malcolm Morgan et al. 2019). OpenTripPlanner calculates the minimum travel time between any specified origin and destination based on underlying transportation networks and schedules. For our analysis, we input the geographic coordinates of each household and workplace as origins, and all essential service facilities within a 30‐mile radius as destinations. OpenTripPlanner's API then returned the estimated travel times by car, walking, and public transit for each origin–destination pair, using up‐to‐date map and transit schedule data from General Transit Feed Specification (GTFS). We obtained the Florida road network from OpenStreetMap (OpenStreetMap contributors 2023). We sourced transit route and schedule data from GTFS files from Collier Area Transit (Florida Transit Data Exchange‐FTIS: Florida Transit Information System n.d.), LeeTran (Transitland 2023), Miami‐Dade Transit (Florida Transit Data Exchange‐FTIS: Florida Transit Information System n.d.), and City of Key West (OpenMobilityData 2023). Note, Hendry County did not have an operating fixed‐route system in 2017. For each household (or workplace) and facility pair, we determined the travel time at 12:00 p.m. on a Wednesday. For transit, we also determined travel times for 12:15 p.m., 12:30 p.m., and 12:45 p.m. to account for fixed schedules. We allowed up to a mile of walking for transit riders and required a minimum of 2 min to allow for any transfers. We also calculated the travel time between each user's home and workplace. From this extracted travel time data, we calculate the minimum travel time by each mode to each service type and the number of each type of essential services facility that is available within 10, 20, 30, 40, 50, and 60 min from each home and workplace. These potential access metrics are computed each day of the study period, tracking the evolution of travel time to the nearest service and the number of available services for the days leading up to and following the disruptive event.

### 4.4 Statistical Modeling

In order to evaluate the importance of access to essential services and recovery, we built out a data set where each observation represents 1 day for each user's home and work location in the month of September. The dependent variable representing each user location's state of recovery is denoted as the binary variable described in Section 4.1, with 1 marking a user location as “unrecovered” and 0 designating “recovered,” including the days pre‐disruption. Features included the travel time to the nearest facility by each mode and the total number of each facility type by each mode within 10, 20, 30, 40, 50, and 60 min. We also generated a variable to represent the pre‐landfall travel time and for the difference between the travel time to a facility on a given day and the pre‐landfall travel time. Other variables include the travel time between the user's home and workplace, days from landfall to represent timing relative to the disruptive event (with positive values representing the days preceding landfall and negative values representing days following landfall), whether public schools in that county were closed, the day of week, whether the location was home or work, the density of the census tract, whether the location was defined as urban or not based on the Florida DOT classification (FDOT Open Data Hub 2024), the evacuation status of the user based on Florida evacuation zones and orders (Florida Division of Emergency Management n.d), and the county (Monroe, Collier, Lee, and Hendry). We also included variables for the previous day's state for that user and the state of the user's other location (home or work). We obtained power outage data from Newspapers (n.d.) to determine variables representing each county's service level at noon, as well as variables for time until power was restored for 25%, 50%, 75%, 90%, 95%, and 99% of the population in units of days and hours. We also included daily county‐level cellular outage data (in %) based on the FCC's Hurricane Irma communications status reports (noa n.d.). We obtained variables for each location's wind exposure in terms of maximum gust wind speed, maximum sustained wind speed, gust duration, sustained duration, and maximum 3‐s gust wind speeds from Brooke Anderson et al. (2018). We also obtained storm surge estimates for Collier and Monroe Counties in the form of variables representing maximum flow speed, maximum significant wave height, maximum force per length, maximum unit discharge, and maximum depth from Appendix A. Finally, we incorporated variables from the CDC's 2018 SoVI (Cutter et al. 2003; CDC/ATSDR Social Vulnerability Index 2023) including raw values as well as percentage estimates, percentile estimates (relative to all census tracts in the United States),“flag” variables for denoting the variable is the 90th percentile, and aggregate “rank” variables that aggregate percentile scores across the themes: socioeconomic, household composition/disability, minority status/language, and housing type/transportation. A table of all of the included variables along with their variable name, units, and source is included in Appendix H along with the associated correlation matrix.

To model the relationship between the aforementioned variables and the state of recovery, we opted to develop a random forest model. Random forest classification is an ensemble decision‐tree–based modeling method that involves estimating the outcome from “majority vote” determined by n individual trees. Each individual tree is developed from a random bootstrap sample of observations from the data set, then for each branch the random subset of features is selected and the optimal split feature is selected from that subset. Random forests are known to be highly accurate and highly interpretable. While they do not output a coefficient estimate for each input variable, they can produce the importance of individual variables relative to the other features in addition to partial dependence plots, which show the overarching relationship of each feature with the predicted outcome. Random forests are well suited for high dimensionality data as they account for high correlation and are robust to overfitting due to their inherent random selection involved in selecting observations and features for each tree. As this data set includes 373 variables and many of the access variables are highly correlated with each other, a random forest is advantageous for directly comparing the relative contribution of individual features based on the objective measure of mean decrease of impurity. This enables us to assess the importance of each individual variable for predicting recovery.

To develop a random forest model, we used a random holdout method for cross‐validation. For each model scenario, we trained the model on 90% of the data (training set), then evaluated its performance on the other 10% of the data (testing set) using outputs for accuracy, precision, recall, AUC‐ROC, F1, and brier‐score as well as a confusion matrix and variable importance ranking. We repeated this 10 times, where the assignment of the data to the training or testing set was random each time. We compared model scenarios based on the average across the 10 iterations.

As we only have storm surge estimates for Collier and Monroe counties, some tested scenarios include only those counties. Other scenarios include Lee and Hendry counties, as well, but exclude the storm surge variables. Because the observed recovery states data are zero‐inflated (about five times as many 0s as 1s), we also tested various class balancing methods including manually weighting the 1s observations by five or by 25, and a balanced subsampling method, which balances the class observation each tree's random selection of observations. Finally, we evaluated the inclusion of different variable combinations. In some scenarios we removed all variables representing the number of facilities within 10, 20, 30, 40, 50, and 60 min as they are highly correlated with each other and with the travel time measures. We also experimented with removing the power variables for restoration times up to some % of the population time, as they were highly correlated with the variable representing % outage at noon. In some scenarios, we removed all transit variables, as transit access was fairly limited across most users and did not include transit service closures. Finally, in some scenarios we removed the aggregated SoVI values, including percentiles and flags as well as any raw values that were redundant of percentage estimates. These scenarios are cataloged in the Appendix along with the output evaluation measures.

## Conflicts of Interest

The authors declare no conflicts of interest.

## Figures and Tables

**FIGURE B.1:**
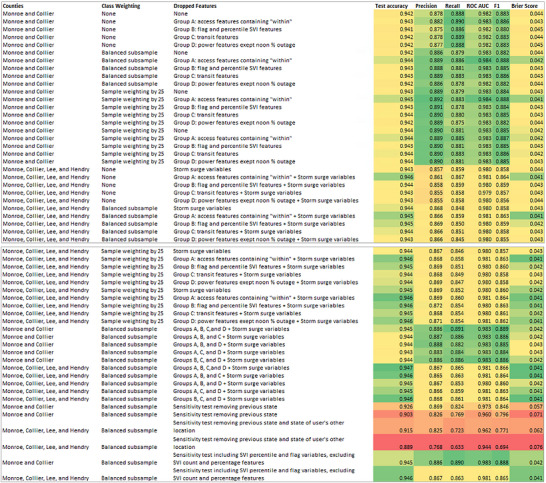
Random forest scenarios with mean of cross‐validation evaluation metrics.

**FIGURE D.1:**
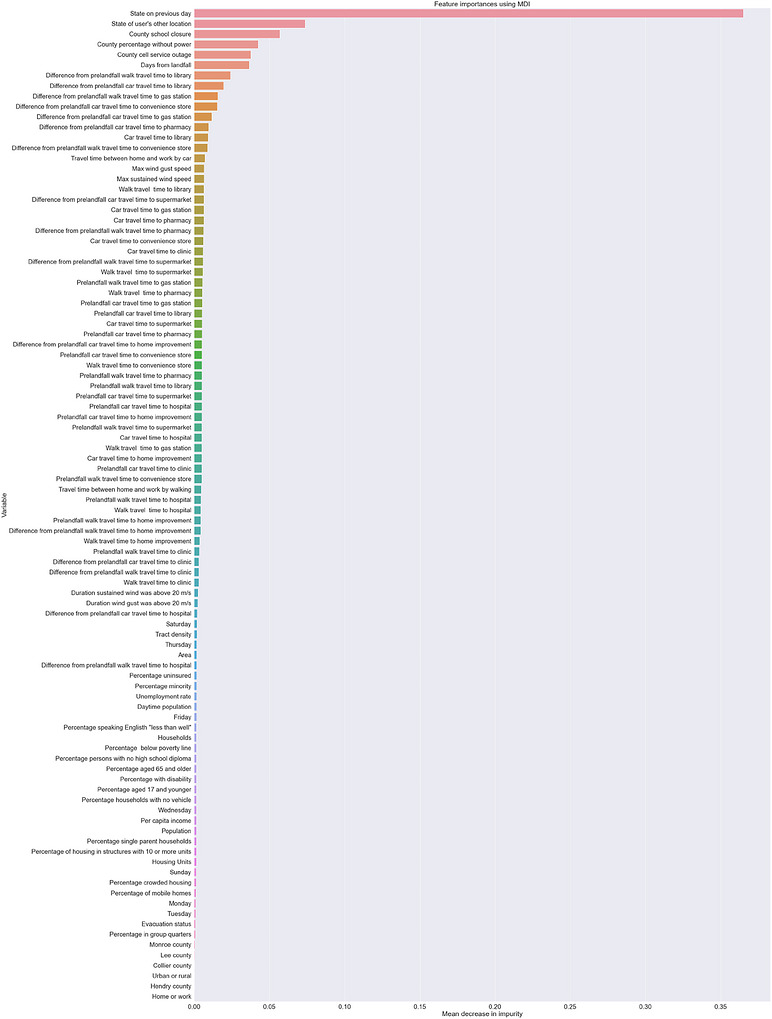
Importance for random forest model including only home locations.

**FIGURE D.2:**
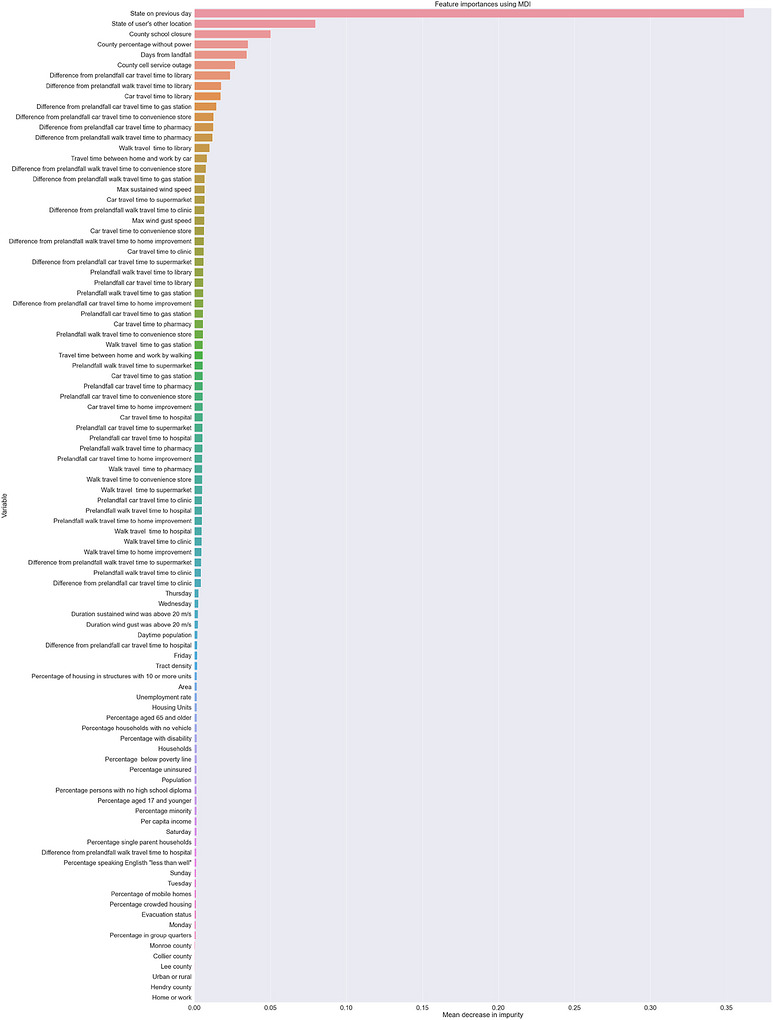
Importance for random forest model including only work locations.

**FIGURE E.1:**
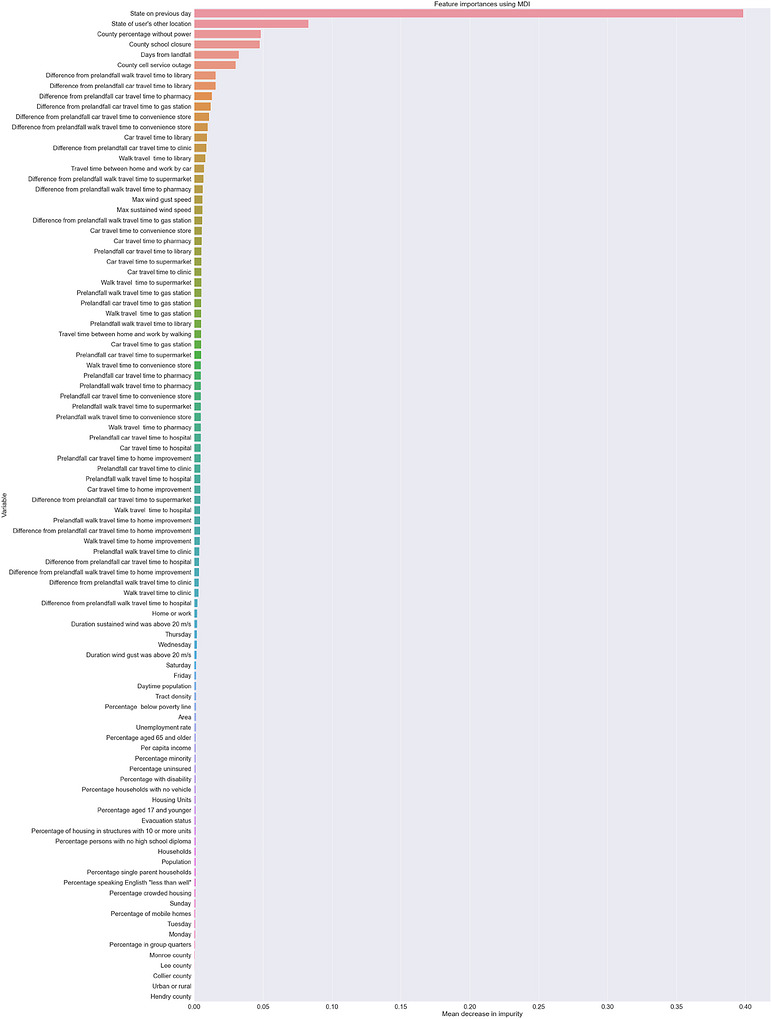
Importance for random forest model including only users with urban home locations.

**FIGURE E.2:**
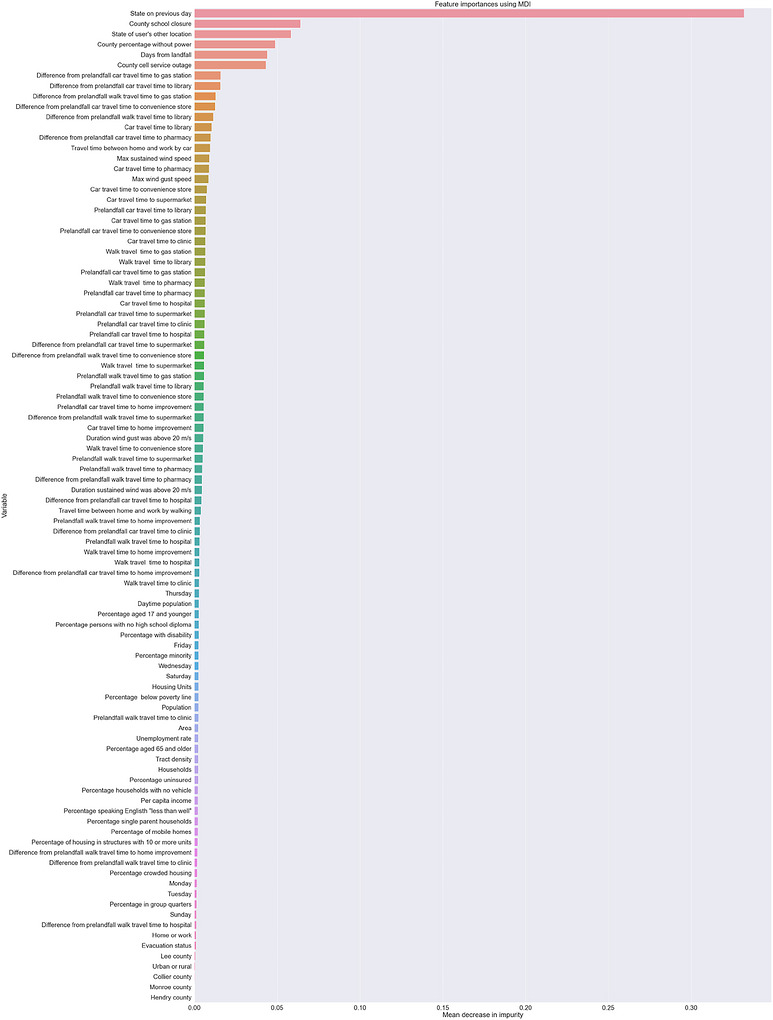
Importance for random forest model including only users with rural home locations.

**FIGURE F.1:**
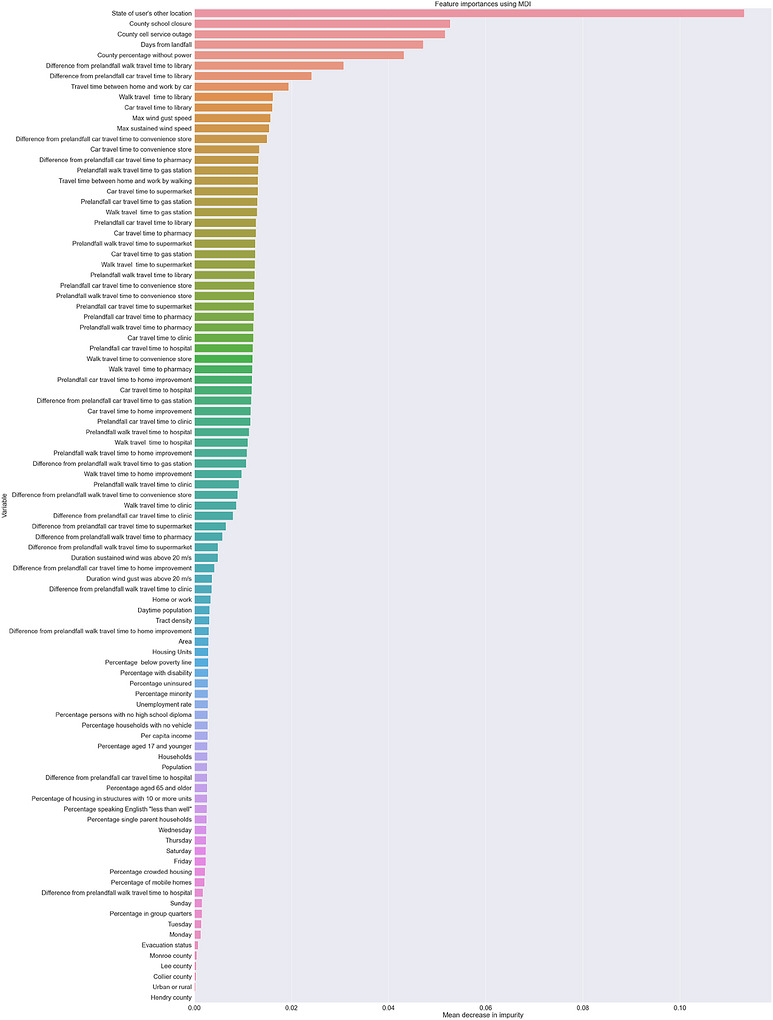
Importance for random forest model including Monroe, Collier, Lee, and Hendry counties and excluding previous state feature.

**FIGURE F.2:**
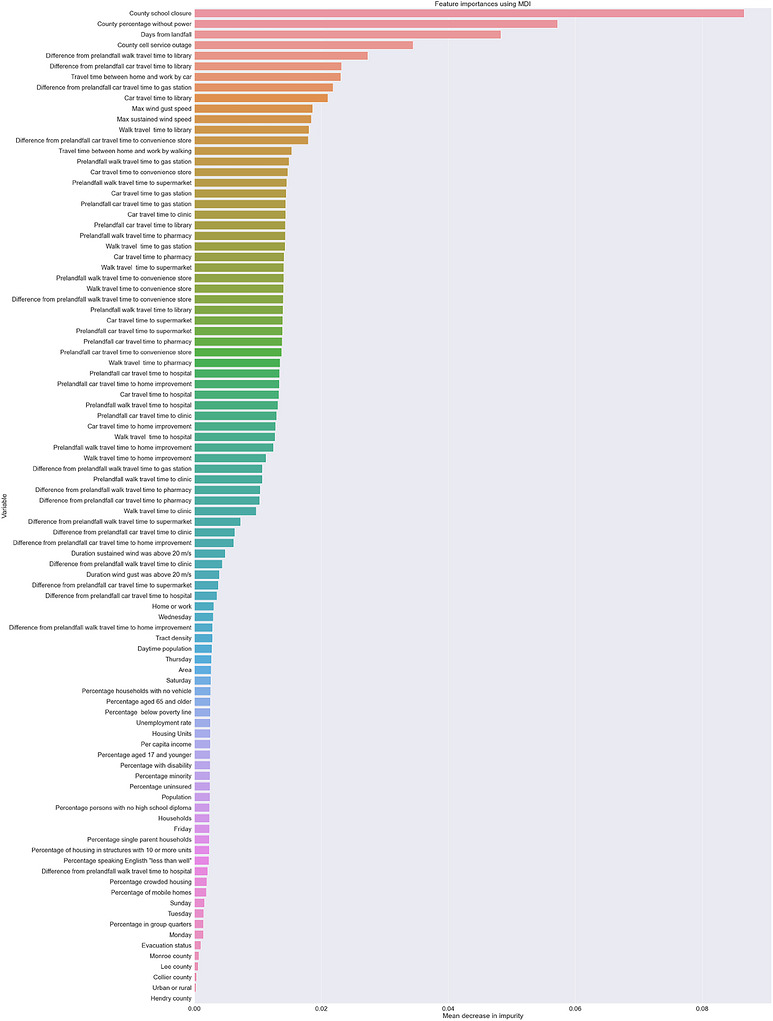
Importance for random forest model including Monroe, Collier, Lee, and Hendry counties and excluding previous state and other location state features.

**FIGURE G.1:**
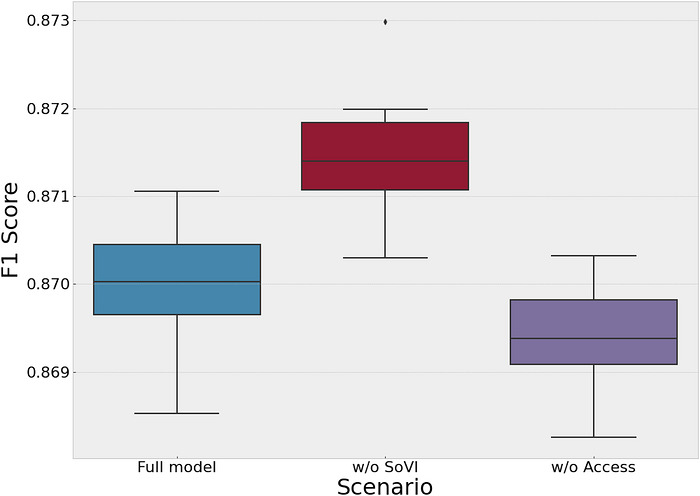
Comparison of F1 performance for models with and without SoVI and access variables.

**FIGURE H.1:**
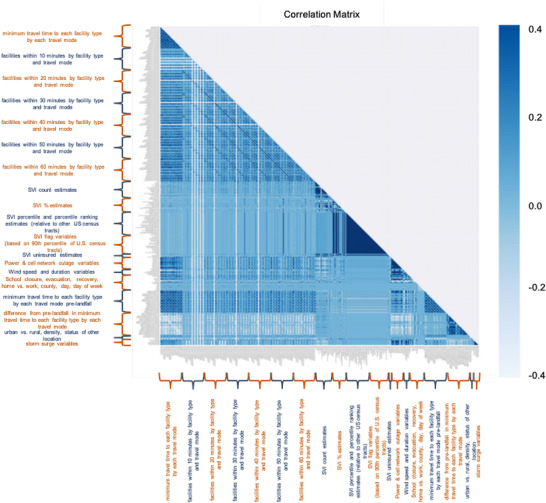
Correlation between model features.

## Appendix A Storm Surge Calculations

To model the storm tide and determine the hydrodynamics each location experienced as a result of Hurricane Irma, the Delft3D Flexible Mesh (Delft3D‐FM) modeling suite is used. Within Delft3D‐FM, the D‐Flow Flexible Mesh (D‐Flow FM) module is coupled with the D‐Waves module. The D‐Flow FM module simulates the hydrodynamics associated with astronomical and meteorological forcings, producing the combined water level (storm tide) due to tides and storm surge (Deltares 2022a). D‐Waves models the generation and evolution of short‐crested waves by utilizing SWAN (Simulating WAves Nearshore) (Deltares 2022b). To couple these modules together, the hydrodynamics simulated in D‐Flow FM, such as water depths and velocities, are used as inputs in D‐Waves, which then provides radiation stresses to D‐Flow FM. An online coupling is used, meaning data between D‐Flow FM and D‐Waves are exchanged every timestep.

Forcing D‐Flow FM requires tidal boundary conditions and meteorological conditions. Tidal boundary conditions for the model are derived from tidal constituents, provided by the Oregon State University Tidal Inversion Software (OTIS) (Egbert and Erofeeva 2002). Wind and pressure fields make up the necessary meteorological forcings, which are generated from best track data of Hurricane Irma from the National Hurricane Center's revised Atlantic hurricane database (HURDAT2) (Landsea and Franklin 2013). This data set is supplemented by the Tropical Cyclone Extended Best Track Data set (EBTRK), providing data on the radius to maximum winds for Irma (Demuth et al. 2006). The Holland model (Holland 2008; Holland et al. 2010) and radius to maximum winds relation (Nederhoff et al. 2019) are then used with these data sets to create the wind and pressure fields at 10 m above sea level to drive the model. This procedure is streamlined by utilizing Delft Dashboard (Van Ormondt et al. 2020).

The model domain is a rectangular area that covers most of Florida, spanning from 22.74 N to 30.94 N and 78.91 W to 84.22 W. The mesh for the D‐Flow FM model is a single, unstructured grid that has a coarse resolution of 10 km and is refined to 80 m in Collier and Monroe counties where there were LBS data and flooding due to Irma. D‐Waves requires nested structured grids to simulate wave parameters, with the coarsest grid having a 10 km resolution and the finest grids (150 m) being located at the same areas of refinement for the D‐Flow FM mesh.

Elevation data sets are retrieved from the NOAA National Centers for Environmental Information's (NCEI) Digital Elevation Model (DEM) Global Mosaic and from the General Bathymetric Chart of the Oceans (GEBCO). GEBCO provides a 15 arcsec resolution elevation data set (GEBCO Compilation Group 2023), which is used for the coarser areas of the model domain. The NCEI DEM Global Mosaic data set provides both 3 arcsecond and 1 arcsecond elevation data sets (NOAA National Centers for Environmental Information (NCEI) 2022), which are used for the areas of refinement in the model domain.

From the model setup described, the following outputs were collected: depth, flow speed, significant wave height, unit discharge, and force per length. The first three parameters (depth, flow speed, and significant wave height) were taken directly from the model output, while the other two parameters (unit discharge and force per length) were calculated from the first three parameters. Unit discharge is simply the flow speed multiplied by the depth. Force per length is calculated with

$$\text{Force per Length}=\rho *d*u^{2},$$

where ρ is the density of water, d is the depth, and u is the flow speed. For each home and work location, the maximum of each of these five hydrodynamic parameters was determined by matching a location's coordinates with the nearest grid cell in the model. This ultimately provided the maximum depth, flow speed, significant wave height, unit discharge, and force per length modeled at each home and work location for Hurricane Irma.

## Appendix B Random Forest Cross‐Validation Scenarios and Results

The table in Figure B.1 shows each model scenario that was evaluated with random sample cross‐validation. For each evaluation metric's column, green indicates better performing score while red indicates poorer performance relative to the rest of the column. Cross‐validation was iterated 10 times with a test set size of 10%.

The confusion matrices across each model iteration revealed the main discrepancy between values was their accuracy with predicting 1 values, as all models predicted “recovered” (0) correctly about 96% of the time, while predictions for “recovering” (1) were correct between 86% and 89% of the time. This slight discrepancy makes sense, given the higher volume and variability of the “recovered” observations (which include pre‐disruption observations) but is still a promising result for predicting “recovering” observations. The models that included only Monroe and Collier counties performed slightly better in terms of ROC‐AUC than those including all four counties, but again the range was small from 0.979 to 0.984. Models that used balanced subsample weighting tended to perform slightly better than the other weighting methods. For both the two‐county and four‐county results, removing all of the designated groups of variables yielded better accuracy for “recovering” predictions as measured by the F1 scores.

## Appendix C Random Forest Results including Storm Surge

To include the modeled storm surge variables, we trained a model using the full data set for just Monroe and Collier counties with balanced subsampling and without the within‐x minutes, transit access, time to % power, and aggregate and percentile SoVI features. The importance ranking of this model is plotted in Figure C.1.

The importances of previous state and the user's other location state show the most predictive importance, followed by county percentage without power, school closures, cell network outages, and days from landfall. These variables are then followed by several access measures denoting the change in access from pre‐event travel times. Wind variables are ranked 19 and 20 followed by many more access variables until evacuation status is 52nd in importance, then down further are variables representing day of week and whether the location was home or work. The storm surge variables all had very low importance, followed by the SoVI values.D.1, D.2, E.1, E.2

## Appendix F Random Forest Sensitivity to Previous and Other Location States

Given the high importance of the previous state and the state of the user's other location for predicting a location's recovery, we wanted to test the sensitivity of the random forest models to these variables. Figure F.1 shows the model for the four counties with all of the same variables as Figure 3 except for “previous state” variable. Figure F.2 shows the model further excluding the “other state” variable. While Figure B.1 shows that these models performed slightly worse than those including these variables, the performance metrics are still very high and the general rankings of the other variables do not change. This affirms the conclusion that access variables consistently rank higher in importance than other social vulnerability measures.

## Appendix G Comparison F1 Scores for Random Forest Models With and Without Access and SoVI Measures

Figure G.1 shows the comparison of F1 scores from the 10 cross‐validation sets for each model. Again, the model including access variables but excluding SoVI variables performed statistically significantly better (*p*‐value < 0.001). The model excluding access variables but including SoVI variables did not have statistically different F1 scores from the full model (*p*‐value = 0.10).

## Appendix H Variables Included in Statistical Modeling for Evaluating Access to Essential Services Versus Recovery

Table H.1 shows all variables used in estimating the recovery status of Floridians in the period surrounding Hurricane Irma. Data were obtained for 7639 total users in Monroe, Collier, Lee, and Hendry counties for each day in September 2017.

**TABLE H.1:** Table of variables used in random forest model estimating recovery state.

Category	Variable name	Variable	Units	Source
Recovery status	Recovery status	Binary	Recovery estimation algorithm	
	State on previous day	Binary	Calculated	
	State of user's other location	Binary	Calculated	
	Home or work	Binary	Home finding algorithm	
Date	Days from landfall	Days	Date	
	Sunday	Binary	Date	
	Monday	Binary	Date	
	Tuesday	Binary	Date	
	Wednesday	Binary	Date	
	Thursday	Binary	Date	
	Friday	Binary	Date	
	Saturday	Binary	Date	
School status	County school closure	Binary	County‐level school districts	
Evacuation status	Evacuation status	Binary	Florida Division of Emergency Management public records request	
Access	Travel time between home and work by car	Minutes	OpenTripPlanner	
	Travel time between home and work by walking	Minutes	OpenTripPlanner	
	Travel time between home and work by transit	Minutes	OpenTripPlanner	
	Car travel time to clinic	Minutes	OpenTripPlanner	
	Transit travel time to clinic	Minutes	OpenTripPlanner	
	Walk travel time to clinic	Minutes	OpenTripPlanner	
	Car travel time to convenience store	Minutes	OpenTripPlanner	
	Transit travel time to convenience store	Minutes	OpenTripPlanner	
	Walk travel time to convenience store	Minutes	OpenTripPlanner	
	Car travel time to gas station	Minutes	OpenTripPlanner	
	Transit travel time to gas station	Minutes	OpenTripPlanner	
	Walk travel time to gas station	Minutes	OpenTripPlanner	
	Car travel time to home improvement	Minutes	OpenTripPlanner	
	Transit travel time to home improvement	Minutes	OpenTripPlanner	
	Walk travel time to home improvement	Minutes	OpenTripPlanner	
	Car travel time to hospital	Minutes	OpenTripPlanner	
	Transit travel time to hospital	Minutes	OpenTripPlanner	
	Walk travel time to hospital	Minutes	OpenTripPlanner	
	Car travel time to library	Minutes	OpenTripPlanner	
	Transit travel time to library	Minutes	OpenTripPlanner	
	Walk travel time to library	Minutes	OpenTripPlanner	
	Car travel time to pharmacy	Minutes	OpenTripPlanner	
	Transit travel time to pharmacy	Minutes	OpenTripPlanner	
	Walk travel time to pharmacy	Minutes	OpenTripPlanner	
	Car travel time to school	Minutes	OpenTripPlanner	
	Transit travel time to school	Minutes	OpenTripPlanner	
	Walk travel time to school	Minutes	OpenTripPlanner	
	Car travel time to supermarket	Minutes	OpenTripPlanner	
	Transit travel time to supermarket	Minutes	OpenTripPlanner	
	Walk travel time to supermarket	Minutes	OpenTripPlanner	
	Pre‐landfall car travel time to clinic	Minutes	OpenTripPlanner	
	Pre‐landfall transit travel time to clinic	Minutes	OpenTripPlanner	
	Pre‐landfall walk travel time to clinic	Minutes	OpenTripPlanner	
	Pre‐landfall car travel time to convenience store	Minutes	OpenTripPlanner	
	Pre‐landfall transit travel time to convenience store	Minutes	OpenTripPlanner	
	Pre‐landfall walk travel time to convenience store	Minutes	OpenTripPlanner	
	Pre‐landfall car travel time to gas station	Minutes	OpenTripPlanner	
	Pre‐landfall transit travel time to gas station	Minutes	OpenTripPlanner	
	Pre‐landfall walk travel time to gas station	Minutes	OpenTripPlanner	
	Pre‐landfall car travel time to home improvement	Minutes	OpenTripPlanner	
	Pre‐landfall transit travel time to home improvement	Minutes	OpenTripPlanner	
	Pre‐landfall walk travel time to home improvement	Minutes	OpenTripPlanner	
	Pre‐landfall car travel time to hospital	Minutes	OpenTripPlanner	
	Pre‐landfall transit travel time to hospital	Minutes	OpenTripPlanner	
	Pre‐landfall walk travel time to hospital	Minutes	OpenTripPlanner	
	Pre‐landfall car travel time to library	Minutes	OpenTripPlanner	
	Pre‐landfall transit travel time to library	Minutes	OpenTripPlanner	
	Pre‐landfall walk travel time to library	Minutes	OpenTripPlanner	
	Pre‐landfall car travel time to pharmacy	Minutes	OpenTripPlanner	
	Pre‐landfall transit travel time to pharmacy	Minutes	OpenTripPlanner	
	Pre‐landfall walk travel time to pharmacy	Minutes	OpenTripPlanner	
	Pre‐landfall car travel time to school	Minutes	OpenTripPlanner	
	Pre‐landfall transit travel time to school	Minutes	OpenTripPlanner	
	Pre‐landfall walk travel time to school	Minutes	OpenTripPlanner	
	Pre‐landfall car travel time to supermarket	Minutes	OpenTripPlanner	
	Pre‐landfall transit travel time to supermarket	Minutes	OpenTripPlanner	
	Pre‐landfall walk travel time to supermarket	Minutes	OpenTripPlanner	
	Difference from pre‐landfall car travel time to clinic	Minutes	OpenTripPlanner	
	Difference from pre‐landfall transit travel time to clinic	Minutes	OpenTripPlanner	
	Difference from pre‐landfall walk travel time to clinic	Minutes	OpenTripPlanner	
	Difference from pre‐landfall car travel time to convenience store	Minutes	OpenTripPlanner	
	Difference from pre‐landfall transit travel time to convenience store	Minutes	OpenTripPlanner	
	Difference from pre‐landfall walk travel time to convenience store	Minutes	OpenTripPlanner	
	Difference from pre‐landfall car travel time to gas station	Minutes	OpenTripPlanner	
	Difference from pre‐landfall transit travel time to gas station	Minutes	OpenTripPlanner	
	Difference from pre‐landfall walk travel time to gas station	Minutes	OpenTripPlanner	
	Difference from pre‐landfall car travel time to home improvement	Minutes	OpenTripPlanner	
	Difference from pre‐landfall transit travel time to home improvement	Minutes	OpenTripPlanner	
	Difference from pre‐landfall walk travel time to home improvement	Minutes	OpenTripPlanner	
	Difference from pre‐landfall car travel time to hospital	Minutes	OpenTripPlanner	
	Difference from pre‐landfall transit travel time to hospital	Minutes	OpenTripPlanner	
	Difference from pre‐landfall walk travel time to hospital	Minutes	OpenTripPlanner	
	Difference from pre‐landfall car travel time to library	Minutes	OpenTripPlanner	
	Difference from pre‐landfall transit travel time to library	Minutes	OpenTripPlanner	
	Difference from pre‐landfall walk travel time to library	Minutes	OpenTripPlanner	
	Difference from pre‐landfall car travel time to pharmacy	Minutes	OpenTripPlanner	
	Difference from pre‐landfall transit travel time to pharmacy	Minutes	OpenTripPlanner	
	Difference from pre‐landfall walk travel time to pharmacy	Minutes	OpenTripPlanner	
	Difference from pre‐landfall car travel time to school	Minutes	OpenTripPlanner	
	Difference from pre‐landfall transit travel time to school	Minutes	OpenTripPlanner	
	Difference from pre‐landfall walk travel time to school	Minutes	OpenTripPlanner	
	Difference from pre‐landfall car travel time to supermarket	Minutes	OpenTripPlanner	
	Difference from pre‐landfall transit travel time to supermarket	Minutes	OpenTripPlanner	
	Difference from pre‐landfall walk travel time to supermarket	Minutes	OpenTripPlanner	
	Number of clinics within 10 min by car	Count	OpenTripPlanner	
	Number of clinics within 10 min by transit	Count	OpenTripPlanner	
	Number of clinics within 10 min by walking	Count	OpenTripPlanner	
	Number of convenience stores within 10 min by car	Count	OpenTripPlanner	
	Number of convenience stores within 10 min by transit	Count	OpenTripPlanner	
	Number of convenience stores within 10 min by walking	Count	OpenTripPlanner	
	Number of gas stations within 10 min by car	Count	OpenTripPlanner	
	Number of gas stations within 10 min by transit	Count	OpenTripPlanner	
	Number of gas stations within 10 min by walking	Count	OpenTripPlanner	
	Number of home improvements within 10 min by car	Count	OpenTripPlanner	
	Number of home improvements within 10 min by transit	Count	OpenTripPlanner	
	Number of home improvements within 10 min by walking	Count	OpenTripPlanner	
	Number of hospitals within 10 min by car	Count	OpenTripPlanner	
	Number of hospitals within 10 min by transit	Count	OpenTripPlanner	
	Number of hospitals within 10 min by walking	Count	OpenTripPlanner	
	Number of libraries within 10 min by car	Count	OpenTripPlanner	
	Number of libraries within 10 min by transit	Count	OpenTripPlanner	
	Number of libraries within 10 min by walking	Count	OpenTripPlanner	
	Number of pharmacies within 10 min by car	Count	OpenTripPlanner	
	Number of pharmacies within 10 min by transit	Count	OpenTripPlanner	
	Number of pharmacies within 10 min by walking	Count	OpenTripPlanner	
	Number of schools within 10 min by car	Count	OpenTripPlanner	
	Number of schools within 10 min by transit	Count	OpenTripPlanner	
	Number of schools within 10 min by walking	Count	OpenTripPlanner	
	Number of supermarkets within 10 min by car	Count	OpenTripPlanner	
	Number of supermarkets within 10 min by transit	Count	OpenTripPlanner	
	Number of supermarkets within 10 min by walking	Count	OpenTripPlanner	
	Number of clinics within 20 min by car	Count	OpenTripPlanner	
	Number of clinics within 20 min by transit	Count	OpenTripPlanner	
	Number of clinics within 20 min by walking	Count	OpenTripPlanner	
	Number of convenience stores within 20 min by car	Count	OpenTripPlanner	
	Number of convenience stores within 20 min by transit	Count	OpenTripPlanner	
	Number of convenience stores within 20 min by walking	Count	OpenTripPlanner	
	Number of gas stations within 20 min by car	Count	OpenTripPlanner	
	Number of gas stations within 20 min by transit	Count	OpenTripPlanner	
	Number of gas stations within 20 min by walking	Count	OpenTripPlanner	
	Number of home improvements within 20 min by car	Count	OpenTripPlanner	
	Number of home improvements within 20 min by transit	Count	OpenTripPlanner	
	Number of home improvements within 20 min by walking	Count	OpenTripPlanner	
	Number of hospitals within 20 min by car	Count	OpenTripPlanner	
	Number of hospitals within 20 min by transit	Count	OpenTripPlanner	
	Number of hospitals within 20 min by walking	Count	OpenTripPlanner	
	Number of libraries within 20 min by car	Count	OpenTripPlanner	
	Number of libraries within 20 min by transit	Count	OpenTripPlanner	
	Number of libraries within 20 min by walking	Count	OpenTripPlanner	
	Number of pharmacies within 20 min by car	Count	OpenTripPlanner	
	Number of pharmacies within 20 min by transit	Count	OpenTripPlanner	
	Number of pharmacies within 20 min by walking	Count	OpenTripPlanner	
	Number of schools within 20 min by car	Count	OpenTripPlanner	
	Number of schools within 20 min by transit	Count	OpenTripPlanner	
	Number of schools within 20 min by walking	Count	OpenTripPlanner	
	Number of supermarkets within 20 min by car	Count	OpenTripPlanner	
	Number of supermarkets within 20 min by transit	Count	OpenTripPlanner	
	Number of supermarkets within 20 min by walking	Count	OpenTripPlanner	
	Number of clinics within 30 min by car	Count	OpenTripPlanner	
	Number of clinics within 30 min by transit	Count	OpenTripPlanner	
	Number of clinics within 30 min by walking	Count	OpenTripPlanner	
	Number of convenience stores within 30 min by car	Count	OpenTripPlanner	
	Number of convenience stores within 30 min by transit	Count	OpenTripPlanner	
	Number of convenience stores within 30 min by walking	Count	OpenTripPlanner	
	Number of gas stations within 30 min by car	Count	OpenTripPlanner	
	Number of gas stations within 30 min by transit	Count	OpenTripPlanner	
	Number of gas stations within 30 min by walking	Count	OpenTripPlanner	
	Number of home improvements within 30 min by car	Count	OpenTripPlanner	
	Number of home improvements within 30 min by transit	Count	OpenTripPlanner	
	Number of home improvements within 30 min by walking	Count	OpenTripPlanner	
	Number of hospitals within 30 min by car	Count	OpenTripPlanner	
	Number of hospitals within 30 min by transit	Count	OpenTripPlanner	
	Number of hospitals within 30 min by walking	Count	OpenTripPlanner	
	Number of libraries within 30 min by car	Count	OpenTripPlanner	
	Number of libraries within 30 min by transit	Count	OpenTripPlanner	
	Number of libraries within 30 min by walking	Count	OpenTripPlanner	
	Number of pharmacies within 30 min by car	Count	OpenTripPlanner	
	Number of pharmacies within 30 min by transit	Count	OpenTripPlanner	
	Number of pharmacies within 30 min by walking	Count	OpenTripPlanner	
	Number of schools within 30 min by car	Count	OpenTripPlanner	
	Number of schools within 30 min by transit	Count	OpenTripPlanner	
	Number of schools within 30 min by walking	Count	OpenTripPlanner	
	Number of supermarkets within 30 min by car	Count	OpenTripPlanner	
	Number of supermarkets within 30 min by transit	Count	OpenTripPlanner	
	Number of supermarkets within 30 min by walking	Count	OpenTripPlanner	
	Number of clinics within 40 min by car	Count	OpenTripPlanner	
	Number of clinics within 40 min by transit	Count	OpenTripPlanner	
	Number of clinics within 40 min by walking	Count	OpenTripPlanner	
	Number of convenience stores within 40 min by car	Count	OpenTripPlanner	
	Number of convenience stores within 40 min by transit	Count	OpenTripPlanner	
	Number of convenience stores within 40 min by walking	Count	OpenTripPlanner	
	Number of gas stations within 40 min by car	Count	OpenTripPlanner	
	Number of gas stations within 40 min by transit	Count	OpenTripPlanner	
	Number of gas stations within 40 min by walking	Count	OpenTripPlanner	
	Number of home improvements within 40 min by car	Count	OpenTripPlanner	
	Number of home improvements within 40 min by transit	Count	OpenTripPlanner	
	Number of home improvements within 40 min by walking	Count	OpenTripPlanner	
	Number of hospitals within 40 min by car	Count	OpenTripPlanner	
	Number of hospitals within 40 min by transit	Count	OpenTripPlanner	
	Number of hospitals within 40 min by walking	Count	OpenTripPlanner	
	Number of libraries within 40 min by car	Count	OpenTripPlanner	
	Number of libraries within 40 min by transit	Count	OpenTripPlanner	
	Number of libraries within 40 min by walking	Count	OpenTripPlanner	
	Number of pharmacies within 40 min by car	Count	OpenTripPlanner	
	Number of pharmacies within 40 min by transit	Count	OpenTripPlanner	
	Number of pharmacies within 40 min by walking	Count	OpenTripPlanner	
	Number of schools within 40 min by car	Count	OpenTripPlanner	
	Number of schools within 40 min by transit	Count	OpenTripPlanner	
	Number of schools within 40 min by walking	Count	OpenTripPlanner	
	Number of supermarkets within 40 min by car	Count	OpenTripPlanner	
	Number of supermarkets within 40 min by transit	Count	OpenTripPlanner	
	Number of supermarkets within 40 min by walking	Count	OpenTripPlanner	
	Number of clinics within 50 min by car	Count	OpenTripPlanner	
	Number of clinics within 50 min by transit	Count	OpenTripPlanner	
	Number of clinics within 50 min by walking	Count	OpenTripPlanner	
	Number of convenience stores within 50 min by car	Count	OpenTripPlanner	
	Number of convenience stores within 50 min by transit	Count	OpenTripPlanner	
	Number of convenience stores within 50 min by walking	Count	OpenTripPlanner	
	Number of gas stations within 50 min by car	Count	OpenTripPlanner	
	Number of gas stations within 50 min by transit	Count	OpenTripPlanner	
	Number of gas stations within 50 min by walking	Count	OpenTripPlanner	
	Number of home improvements within 50 min by car	Count	OpenTripPlanner	
	Number of home improvements within 50 min by transit	Count	OpenTripPlanner	
	Number of home improvements within 50 min by walking	Count	OpenTripPlanner	
	Number of hospitals within 50 min by car	Count	OpenTripPlanner	
	Number of hospitals within 50 min by transit	Count	OpenTripPlanner	
	Number of hospitals within 50 min by walking	Count	OpenTripPlanner	
	Number of libraries within 50 min by car	Count	OpenTripPlanner	
	Number of libraries within 50 min by transit	Count	OpenTripPlanner	
	Number of libraries within 50 min by walking	Count	OpenTripPlanner	
	Number of pharmacies within 50 min by car	Count	OpenTripPlanner	
	Number of pharmacies within 50 min by transit	Count	OpenTripPlanner	
	Number of pharmacies within 50 min by walking	Count	OpenTripPlanner	
	Number of schools within 50 min by car	Count	OpenTripPlanner	
	Number of schools within 50 min by transit	Count	OpenTripPlanner	
	Number of schools within 50 min by walking	Count	OpenTripPlanner	
	Number of supermarkets within 50 min by car	Count	OpenTripPlanner	
	Number of supermarkets within 50 min by transit	Count	OpenTripPlanner	
	Number of supermarkets within 50 min by walking	Count	OpenTripPlanner	
	Number of clinics within 60 min by car	Count	OpenTripPlanner	
	Number of clinics within 60 min by transit	Count	OpenTripPlanner	
	Number of clinics within 60 min by walking	Count	OpenTripPlanner	
	Number of convenience stores within 60 min by car	Count	OpenTripPlanner	
	Number of convenience stores within 60 min by transit	Count	OpenTripPlanner	
	Number of convenience stores within 60 min by walking	Count	OpenTripPlanner	
	Number of gas stations within 60 min by car	Count	OpenTripPlanner	
	Number of gas stations within 60 min by transit	Count	OpenTripPlanner	
	Number of gas stations within 60 min by walking	Count	OpenTripPlanner	
	Number of home improvements within 60 min by car	Count	OpenTripPlanner	
	Number of home improvements within 60 min by transit	Count	OpenTripPlanner	
	Number of home improvements within 60 min by walking	Count	OpenTripPlanner	
	Number of hospitals within 60 min by car	Count	OpenTripPlanner	
	Number of hospitals within 60 min by transit	Count	OpenTripPlanner	
	Number of hospitals within 60 min by walking	Count	OpenTripPlanner	
	Number of libraries within 60 min by car	Count	OpenTripPlanner	
	Number of libraries within 60 min by transit	Count	OpenTripPlanner	
	Number of libraries within 60 min by walking	Count	OpenTripPlanner	
	Number of pharmacies within 60 min by car	Count	OpenTripPlanner	
	Number of pharmacies within 60 min by transit	Count	OpenTripPlanner	
	Number of pharmacies within 60 min by walking	Count	OpenTripPlanner	
	Number of schools within 60 min by car	Count	OpenTripPlanner	
	Number of schools within 60 min by transit	Count	OpenTripPlanner	
	Number of schools within 60 min by walking	Count	OpenTripPlanner	
	Number of supermarkets within 60 min by car	Count	OpenTripPlanner	
	Number of supermarkets within 60 min by transit	Count	OpenTripPlanner	
	Number of supermarkets within 60 min by walking	Count	OpenTripPlanner	
Geography	Area	Square miles (census tract)	CDC 2018 SVI	
	Urban or rural	Binary	TDA Functional Classification and Urban Boundary	
	Monroe county	Binary	TIGER/Line Florida 2017	
	Collier county	Binary	TIGER/Line Florida 2018	
	Lee county	Binary	TIGER/Line Florida 2019	
	Hendry county	Binary	TIGER/Line Florida 2020	
Social vulnerability	Population	Count (census tract)	CDC 2018 SVI	
	Housing Units	Count (census tract)	CDC 2018 SVI	
	Households	Count (census tract)	CDC 2018 SVI	
	Persons below poverty	Count (census tract)	CDC 2018 SVI	
	Civilians(age 16+) unemployed	Count (census tract)	CDC 2018 SVI	
	Per capita income	$ (census tract)	CDC 2018 SVI	
	Persons (age 25+) with no high school diploma	Count (census tract)	CDC 2018 SVI	
	Persons aged 65 and older	Count (census tract)	CDC 2018 SVI	
	Persons aged 17 and younger	Count (census tract)	CDC 2018 SVI	
	Civilian noninstitutionalized population with a disability	Count (census tract)	CDC 2018 SVI	
	Single parent household with children under 18	Count (census tract)	CDC 2018 SVI	
	“Minority (all persons except white, non ‐ Hispanic)”	Count (census tract)	CDC 2018 SVI	
	“Persons (age 5+) who speak English ‘less than well”'	Count (census tract)	CDC 2018 SVI	
	Housing in structures with 10 or more units	Count (census tract)	CDC 2018 SVI	
	Mobile homes	Count (census tract)	CDC 2018 SVI	
	“At household level (occupied housing units), more people than rooms”	Count (census tract)	CDC 2018 SVI	
	Households with no vehicle available	Count (census tract)	CDC 2018 SVI	
	Persons in group quarters	Count (census tract)	CDC 2018 SVI	
	Percentage below poverty line	% (of census tract)	CDC 2018 SVI	
	Unemployment rate	% (of census tract)	CDC 2018 SVI	
	Percentage persons with no high school diploma	% (of census tract)	CDC 2018 SVI	
	Percentage aged 65 and older	% (of census tract)	CDC 2018 SVI	
	Percentage aged 17 and younger	% (of census tract)	CDC 2018 SVI	
	Percentage with disability	% (of census tract)	CDC 2018 SVI	
	Percentage single parent households	% (of census tract)	CDC 2018 SVI	
	Percentage minority	% (of census tract)	CDC 2018 SVI	
	“Percentage speaking English ‘less than well”'	% (of census tract)	CDC 2018 SVI	
	Percentage of housing in structures with 10 or…	% (of census tract)	CDC 2018 SVI	
	Percentage of mobile homes	% (of census tract)	CDC 2018 SVI	
	Percentage crowded housing	% (of census tract)	CDC 2018 SVI	
	Percentage households with no vehicle	% (of census tract)	CDC 2018 SVI	
	Percentage in group quarters	% (of census tract)	CDC 2018 SVI	
	Percentile percentage of persons below poverty	Percentile (census tract relative to other US census tracts)	CDC 2018 SVI	
	Percentile percentage of civilian (age 16+) unemployed	Percentile (census tract relative to other US census tracts)	CDC 2018 SVI	
	Percentile per capita income	Percentile (census tract relative to other US census tracts)	CDC 2018 SVI	
	Percentile percentage of persons with no high school diploma (age 25+)	Percentile (census tract relative to other US census tracts)	CDC 2018 SVI	
	Socioeconomic theme: EPL_POV + EPL_UNEMP + EPL_PCI + EPL_NOHSDP	Sum of % (census tract)	CDC 2018 SVI	
	Percentile ranking for socioeconomic theme	Percent rank (census tract relative to other U.S. census tracts)	CDC 2018 SVI	
	Percentile percentage of persons aged 65 and older	Percentile (census tract relative to other U.S. census tracts)	CDC 2018 SVI	
	Percentile percentage of persons aged 17 and younger	Percentile (census tract relative to other U.S. census tracts)	CDC 2018 SVI	
	Percentile percentage of civilian noninstitutionalized population with a disability	Percentile (census tract relative to other U.S. census tracts)	CDC 2018 SVI	
	Percentile percentage of single parent households with children under 18	Percentile (census tract relative to other U.S. census tracts)	CDC 2018 SVI	
	Household composition theme: EPL_AGE65 + EPL_AGE17 + EPL_DISABL + EPL_SNGPNT	Sum of % (census tract)	CDC 2018 SVI	
	Percentile ranking for household composition theme	Percent rank (census tract relative to other U.S. census tracts)	CDC 2018 SVI	
	“Percentile percentage minority (all persons except white, non ‐ Hispanic)”	Percentile (census tract relative to other U.S. census tracts)	CDC 2018 SVI	
	“Percentile percentage of persons (age 5+) who speak English ‘less than well”'	Percentile (census tract relative to other U.S. census tracts)	CDC 2018 SVI	
	Minority status/language theme: EPL_MINRTY + EPL_LIMENG	Sum of % (census tract)	CDC 2018 SVI	
	Percentile ranking for minority status/language theme	Percentile (census tract relative to other U.S. census tracts)	CDC 2018 SVI	
	Percentile percentage housing in structures with 10 or more units	Percentile (census tract relative to other U.S. census tracts)	CDC 2018 SVI	
	Percentile percentage mobile homes	Percentile (census tract relative to other U.S. census tracts)	CDC 2018 SVI	
	Percentile percentage households with more people than rooms	Percentile (census tract relative to other U.S. census tracts)	CDC 2018 SVI	
	Percentile percentage households with no vehicle available	Percentile (census tract relative to other U.S. census tracts)	CDC 2018 SVI	
	Percentile percentage of persons in group quarters estimate	Percentile (census tract relative to other U.S. census tracts)	CDC 2018 SVI	
	Housing type/transportation theme: EPL_MUNIT + EPL_MOBIL + EPL_CROWD + EPL_NOVEH + EPL_GROUPQ	Sum of % (census tract)	CDC 2018 SVI	
	Percentile ranking for housing type / transportation theme	Percent rank (census tract relative to other U.S. census tracts)	CDC 2018 SVI	
	Sum of series themes: SPL_THEME1 + SPL_THEME2 + SPL_THEME3 + SPL_THEME4	Sum of % (census tract)	CDC 2018 SVI	
	Percentile ranking for sum of series themes	Percent rank (census tract relative to other U.S. census tracts)	CDC 2018 SVI	
	Flag ‐ percentage of persons in poverty is in the 90th percentile	Binary	CDC 2018 SVI	
	Flag ‐ the percentage of civilian unemployed is in the 90th percentile	Binary	CDC 2018 SVI	
	Flag ‐ per capita income is in the 90th percentile	Binary	CDC 2018 SVI	
	Flag ‐ the percentage of persons with no high school diploma is in the 90th percentile	Binary	CDC 2018 SVI	
	Sum of flags for socioeconomic status theme: F_POV + F_UNEMP + F_PCI + F_NOHSDP	Count	CDC 2018 SVI	
	Flag ‐ the percentage of persons aged 65 and older is in the 90th percentile	Binary	CDC 2018 SVI	
	Flag ‐ the percentage of persons aged 17 and younger is in the 90th percentile	Binary	CDC 2018 SVI	
	Flag ‐ the percentage of persons with a disability is in the 90th percentile	Binary	CDC 2018 SVI	
	Flag ‐ the percentage of single parent households is in the 90th percentile	Binary	CDC 2018 SVI	
	Sum of flags for household composition theme: F_AGE65 + F_AGE17 + F_DISABL + F_SNGPNT	Count	CDC 2018 SVI	
	Flag ‐ the percentage of minority is in the 90th percentile	Binary	CDC 2018 SVI	
	Flag ‐ the percentage those with limited English is in the 90th percentile	Binary	CDC 2018 SVI	
	Sum of flags for Minority Status/Language theme	Count	CDC 2018 SVI	
	Flag ‐ the percentage of households in multi ‐unit housing is in the 90th percentile	Binary	CDC 2018 SVI	
	Flag ‐ the percentage of mobile homes is in the 90th percentile	Binary	CDC 2018 SVI	
	Flag ‐ the percentage of crowded households is in the 90th percentile	Binary	CDC 2018 SVI	
	Flag ‐ the percentage of households with no vehicles is in the 90th percentile	Binary	CDC 2018 SVI	
	Flag ‐ the percentage of persons in institutionalized group quarters is in the 90th percentile	Binary	CDC 2018 SVI	
	Sum of flags for housing type / transportation theme	Count	CDC 2018 SVI	
	Sum of flags for the four themes	Count	CDC 2018 SVI	
	Uninsured in the total civilian noninstitutionalized population	Count (census tract)	CDC 2018 SVI	
	Percentage uninsured	% (of census tract)	CDC 2018 SVI	
	Daytime population	Count (census tract)	CDC 2018 SVI	
	Tract density	Population/square mile (census tract)	Calculated from SVI variables	
Power	Tract percentage without power	% (of county)	Florida Division of Emergency Management as reported through Florida Today	
	Days until power restored for 25% of county	Days	Florida Division of Emergency Management as reported through Florida Today	
	Days until power restored for 50% of county	Days	Florida Division of Emergency Management as reported through Florida Today	
	Days until power restored for 75% of county	Days	Florida Division of Emergency Management as reported through Florida Today	
	Days until power restored for 90% of county	Days	Florida Division of Emergency Management as reported through Florida Today	
	Days until power restored for 95% of county	Days	Florida Division of Emergency Management as reported through Florida Today	
	Days until power restored for 99% of county	Days	Florida Division of Emergency Management as reported through Florida Today	
	Hours until power restored for 25% of county	Hours	Florida Division of Emergency Management as reported through Florida Today	
	Hours until power restored for 50% of county	Hours	Florida Division of Emergency Management as reported through Florida Today	
	Hours until power restored for 75% of county	Hours	Florida Division of Emergency Management as reported through Florida Today	
	Hours until power restored for 90% of county	Hours	Florida Division of Emergency Management as reported through Florida Today	
	Hours until power restored for 95% of county	Hours	Florida Division of Emergency Management as reported through Florida Today	
	Hours until power restored for 99% of county	Hours	Florida Division of Emergency Management as reported through Florida Today	
Cell network	County cell service outage	% (of county)	FCC Hurricane Irma Communications Status Reports	
Wind	Max wind gust speed	Meters/second	(Brooke Anderson et al. 2018)	
	Max sustained wind speed	Meters/second	(Brooke Anderson et al. 2018)	
	Duration wind gust was above 20 m/s	Hours	(Brooke Anderson et al. 2018)	
	Duration sustained wind was above 20 m/s	Hours	(Brooke Anderson et al. 2018)	
Storm surge	Max storm surge depth	Meters	Appendix A	
	Max storm surge flow speed	Meters/second	Appendix A	
	Max storm surge unit discharge	Square‐meters/second	Appendix A	
	Max storm surge force per length	Kilograms/square‐second	Appendix A	
	Max storm surge wave height	Meters	Appendix A	

Figure H.1 shows the correlation between the 374 included features. For readability, labels are included to describe groups of features.

In general, correlation between feature pairs is low except for between variables within the same group. The pre‐landfall access values are deduced from the minimum travel time access values, and so are highly correlated. The social vulnerability index percentile and flag features are highly correlated, in part because they do not contain much variability as they are determined relative to other census tract values across the United States. The storm surge variables appear correlated with the wind and power outage variables.

## References

[risa70323-bib-0007] Allenby, B. , and J. Fink . 2005. “Toward Inherently Secure and Resilient Societies.” Science 309: 1034–1036. 16099973 10.1126/science.1111534

[risa70323-bib-0093] American Association of State Highway and Transportation Officials . 2017. “Census Tract Planning Products (CTPP)Overview.” https://ctppdata.transportation.org.

[risa70323-bib-0008] Anderson, S. E. , R. R. Bart , M. C. Kennedy , et al. 2018. “The Dangers of Disaster‐Driven Responses to Climate Change.” Nature Climate Change 8: 651–653.

[risa70323-bib-0009] Aven, T. 2011. “On Some Recent Definitions and Analysis Frameworks for Risk, Vulnerability, and Resilience.” Risk Analysis 31: 515–522. 21077926 10.1111/j.1539-6924.2010.01528.x

[risa70323-bib-0010] Bakkensen, L. A. , C. Fox‐Lent , L. K. Read , and I. Linkov . 2017. “Validating Resilience and Vulnerability Indices in the Context of Natural Disasters.” Risk Analysis 37: 982–1004. 27577104 10.1111/risa.12677

[risa70323-bib-0011] Barrington‐Leigh, C. , and A. Millard‐Ball . 2017. “The World's User‐Generated Road Map Is More Than 80% Complete.” PLOS One 12: e0180698. 28797037 10.1371/journal.pone.0180698 PMC5552279

[risa70323-bib-0012] Beccari, B. 2016. “A Comparative Analysis of Disaster Risk, Vulnerability and Resilience Composite Indicators.” *PLOS Currents* 8. https://currents.plos.org/disasters/article/a‐comparative‐analysis‐of‐disaster‐risk‐vulnerability‐and‐resilience‐composite‐indicators/. 10.1371/currents.dis.453df025e34b682e9737f95070f9b970 PMC4807925 27066298

[risa70323-bib-0013] Bills, T. S. , and J. L. Walker . 2017. “Looking Beyond the Mean for Equity Analysis: Examining Distributional Impacts of Transportation Improvements.” Transport Policy 54: 61–69.

[risa70323-bib-0014] Birkmann, J. 2007. “Risk and Vulnerability Indicators at Different Scales: Applicability, Usefulness and Policy Implications.” Environmental Hazards 7: 20–31.

[risa70323-bib-0015] Boeing, G. 2017. “OSMnx: A Python Package to Work With Graph‐Theoretic OpenStreetMap Street Networks.” Journal Open Source Software 2: 215.

[risa70323-bib-0016] Bolin, R. , and L. Stanford . 1998. “The Northridge Earthquake: Community‐Based Approaches to Unmet Recovery Needs.” Disasters 22: 21–38. 9549171 10.1111/1467-7717.00073

[risa70323-bib-0017] Boustan, L. P. , M. E. Kahn , P. W. Rhode , and M. L. Yanguas . 2020. “The Effect of Natural Disasters on Economic Activity in US Counties: A Century of Data.” Journal of Urban Economics 118: 103257.

[risa70323-bib-0018] Brooke Anderson, G. , A. Schumacher , S. Guikema , J. M. Ferreri , and S. Quiring . 2018. “R Stormwindmodel Package.” https://cran.r‐hub.io/web/packages/stormwindmodel/vignettes/Details.html. Accessed September 19, 2023.

[risa70323-bib-0019] Bruneau, M. , S. E. Chang , R. T. Eguchi , et al. 2003. “A Framework to Quantitatively Assess and Enhance the Seismic Resilience of Communities.” Earthquake Spectra 19: 733–752.

[risa70323-bib-0020] Calabrese, F. , M. Diao , G. Di Lorenzo , J. Ferreira , and C. Ratti . 2013. “Understanding Individual Mobility Patterns from Urban Sensing Data: A Mobile Phone Trace Example.” Transportation Research Part C: Emerging Technologies 26: 301–313.

[risa70323-bib-0004] CDC/ATSDR Social Vulnerability Index . 2023. https://www.atsdr.cdc.gov/placeandhealth/svi/index.html. Accessed September 17, 2023.

[risa70323-bib-0021] Changnon, S. A. , R. A. Pielke , D. Changnon , R. T. Sylves , and R. Pulwarty . 2000. “Human Factors Explain the Increased Losses From Weather and Climate Extremes.” Bulletin of the American Meteorological Society 81: 437–442.

[risa70323-bib-0022] Chen, C. , L. Bian , and J. Ma . 2014. “From Traces to Trajectories: How Well Can We Guess Activity Locations From Mobile Phone Traces?” Transportation Research Part C: Emerging Technologies 46: 326–337.

[risa70323-bib-0023] Chen, C. , J. Ma , Y. Susilo , Y. Liu , and M. Wang . 2016. “The Promises of Big Data and Small Data for Travel Behavior (Aka Human Mobility) Analysis.” Transportation Research Part C: Emerging Technologies 68: 285–299. 27182125 10.1016/j.trc.2016.04.005 PMC4862004

[risa70323-bib-0024] Chen, M. K. , and R. Rohla . 2018. “The Effect of Partisanship and Political Advertising on Close Family Ties.” Science 360: 1020–1024. 29853686 10.1126/science.aaq1433

[risa70323-bib-0025] Cinnamon, J. , S. K. Jones , and W. N. Adger . 2016. “Evidence and Future Potential of Mobile Phone Data for Disease Disaster Management.” Geoforum 75: 253–264. 32287362 10.1016/j.geoforum.2016.07.019 PMC7127132

[risa70323-bib-0026] OpenStreetMap contributors . 2023. Geofabrik [florida‐230101]. Retrieved from https://download.geofabrik.de/north‐america/us/florida.html.

[risa70323-bib-0027] Cutter, S. L. 2016. “The Landscape of Disaster Resilience Indicators in the USA.” Natural Hazards 80: 741–758.

[risa70323-bib-0028] Cutter, S. L. , K. D. Ash , and C. T. Emrich . 2014. “The Geographies of Community Disaster Resilience.” Global Environmental Change 29: 65–77.

[risa70323-bib-0029] Cutter, S. L. , B. J. Boruff , and W. L. Shirley . 2003. “Social Vulnerability to Environmental Hazards.” Social Science Quarterly 84: 242–261.

[risa70323-bib-0030] de Arruda, F. H. , S. M. Reia , S. Ruan , et al. 2024. “An OpenStreetMap derived building classification dataset for the United States.” Scientific Data 11: 1210. 39521802 10.1038/s41597-024-04046-w PMC11550320

[risa70323-bib-0031] Deltares . 2022a. D‐Flow Flexible Mesh User Manual .

[risa70323-bib-0032] Deltares . 2022b. D‐Waves User Manual .

[risa70323-bib-0033] Demuth, J. L. , M. DeMaria , and J. A. Knaff . 2006. “Improvement of Advanced Microwave Sounding Unit Tropical Cyclone Intensity and Size Estimation Algorithms.” Journal of Applied Meteorology and Climatology 45: 1573–1581.

[risa70323-bib-0034] Dobra, A. , N. E. Williams , and N. Eagle . 2015. “Spatiotemporal Detection of Unusual Human Population Behavior Using Mobile Phone Data.” PLoS One 10: e0120449. 25806954 10.1371/journal.pone.0120449 PMC4373934

[risa70323-bib-0035] Eckert, J. , and S. Shetty . 2011. “Food Systems, Planning and Quantifying Access: Using GIS to Plan for Food Retail.” Applied Geography 31: 1216–1223.

[risa70323-bib-0036] Egbert, G. D. , and S. Y. Erofeeva . 2002. “Efficient Inverse Modeling of Barotropic Ocean Tides.” Journal of Atmospheric and Oceanic Technology 19: 183–204.

[risa70323-bib-0037] Elmqvist, T. , E. Andersson , N. Frantzeskaki , et al. 2019. “Sustainability and Resilience for Transformation in the Urban Century.” Nature Sustainability 2: 267–273.

[risa70323-bib-0038] Fan, C. , X. Jiang , R. Lee , and A. Mostafavi . 2022. “Equality of Access and Resilience in Urban Population‐Facility Networks.” npj Urban Sustainability 2: 9.

[risa70323-bib-0039] FDOT Open Data Hub . 2024. “FDOT FHWA Smoothed Urban Boundaries (TDA).” GIS Dataset, FDOT Open Data Hub, Source Spatial Dataset Created November 13, 2024. Accessed July 13, 2026.

[risa70323-bib-0040] Fekete, A. 2012. “Spatial Disaster Vulnerability and Risk Assessments: Challenges in their Quality and Acceptance.” Natural Hazards 61: 1161–1178.

[risa70323-bib-0041] FEMA . n.d. “National Risk Index.” https://hazards.fema.gov/nri/learn‐more. Accessed September 1, 2023.

[risa70323-bib-0042] Finucane, M. L. , J. Acosta , A. Wicker , and K. Whipkey . 2020. “Short‐Term Solutions to a Long‐Term Challenge: Rethinking Disaster Recovery Planning to Reduce Vulnerabilities and Inequities.” *International Journal of Environmental Research and Public Health* 17: 482. 10.3390/ijerph17020482 PMC7013733 31940859

[risa70323-bib-0001] Florida Division of Emergency Management . n.d. https://www.floridadisaster.org/. Accessed October 1, 2023.

[risa70323-bib-0002] Florida Transit Data Exchange‐ FTIS: Florida Transit Information System . n.d. https://ftis.org/Posts.aspx. Accessed September 15, 2023.

[risa70323-bib-0003] Hurricane Irma . n.d. https://www.fcc.gov/irma. Accessed September 17, 2023.

[risa70323-bib-0044] Frazier, T. G. , C. M. Thompson , and R. J. Dezzani . 2014. “A Framework for the Development of the SERV Model: A Spatially Explicit Resilience‐Vulnerability Model.” Applied Geography 51: 158–172.

[risa70323-bib-0045] Frazier, T. G. , C. M. Thompson , R. J. Dezzani , and D. Butsick . 2013. “Spatial and Temporal Quantification of Resilience at the Community Scale.” Applied Geography 42: 95–107.

[risa70323-bib-0046] Ganin, A. A. , M. Kitsak , D. Marchese , J. M. Keisler , T. Seager , and I. Linkov . 2017. “Resilience and Efficiency in Transportation Networks.” Science Advances 3: e1701079. 29291243 10.1126/sciadv.1701079 PMC5744464

[risa70323-bib-0047] Gardoni, P. , and C. Murphy . 2020. “Society‐Based Design: Promoting Societal Well‐Being by Designing Sustainable and Resilient Infrastructure.” Sustainable and Resilient Infrastructure 5: 4–19.

[risa70323-bib-0048] GEBCO Compilation Group . 2023. “GEBCO Gridded Bathymetry Data”.

[risa70323-bib-0049] Godschalk, D. R. 2003. “Urban Hazard Mitigation: Creating Resilient Cities.” Natural Hazards Review 4: 136–143.

[risa70323-bib-0050] Goodman, Z. T. , C. A. Stamatis , J. Stoler , C. T. Emrich , and M. M. Llabre . 2021. “Methodological Challenges to Confirmatory Latent Variable Models of Social Vulnerability.” Natural Hazards 106: 2731–2749. 33612967 10.1007/s11069-021-04563-6 PMC7882037

[risa70323-bib-0051] Green, R. , L. K. Bates , and A. Smyth . 2007. “Impediments to Recovery in New Orleans' Upper and Lower Ninth Ward: One Year After Hurricane Katrina.” Disasters 31: 311–335. 18028156 10.1111/j.1467-7717.2007.01011.x

[risa70323-bib-0052] Gunda, T. , A. Wachtel , S. Khadka Mishra , and E. Moog . 2023. “Quantitative Approaches for Including Equity in Risk and Resilience Infrastructure Planning Analyses.” Risk Analysis 45: 3427–3438. 37772629 10.1111/risa.14230

[risa70323-bib-0053] Gurney, K. , and Miami . 2017. “Some Florida Keys Schools to Open Monday. For Others Hit by Irma, It Could Be Much Longer.” Miami Herald . https://www.miamiherald.com/news/weather/hurricane/article174184716.html.

[risa70323-bib-0054] Hecht, R. , C. Kunze , and S. Hahmann . 2013. “Measuring Completeness of Building Footprints in OpenStreetMap Over Space and Time.” ISPRS International Journal of Geo‐Information 2: 1066–1091.

[risa70323-bib-0055] Hendricks, M. D. , and S. Van Zandt . 2021. “Unequal Protection Revisited: Planning for Environmental Justice, Hazard Vulnerability, and Critical Infrastructure in Communities of Color.” Environmental Justice 14: 87–97.

[risa70323-bib-0056] Henry, D. , and J. Emmanuel Ramirez‐Marquez . 2012. “Generic Metrics and Quantitative Approaches for System Resilience as a Function of Time.” Reliability Engineering and System Safety 99: 114–122.

[risa70323-bib-0057] Holland, G. 2008. “A Revised Hurricane Pressure–Wind Model.” Monthly Weather Review 136: 3432–3445.

[risa70323-bib-0058] Holland, G. J. , J. I. Belanger , and A. Fritz . 2010. “A Revised Model for Radial Profiles of Hurricane Winds.” Monthly Weather Review 138: 4393–4401.

[risa70323-bib-0059] Hong, B. , B. J. Bonczak , A. Gupta , and C. E. Kontokosta . 2021. “Measuring Inequality in Community Resilience to Natural Disasters Using Large‐Scale Mobility Data.” Nature Communications 12: 1870. 10.1038/s41467-021-22160-w PMC7994553 33767142

[risa70323-bib-0060] Hosseini, S. , K. Barker , and J. E. Ramirez‐Marquez . 2016. “A Review of Definitions and Measures of System Resilience.” Reliability Engineering and System Safety 145: 47–61.

[risa70323-bib-0061] Howell, J. , and J. R. Elliott . 2018. “Damages Done: The Longitudinal Impacts of Natural Hazards on Wealth Inequality in the United States.” Social Problems 66: 448–467.

[risa70323-bib-0062] Jones, K. H. , H. Daniels , S. Heys , and D. V. Ford . 2018. “Challenges and Potential Opportunities of Mobile Phone Call Detail Records in Health Research: Review.” JMIR Mhealth Uhealth 6: e161. 30026176 10.2196/mhealth.9974 PMC6072975

[risa70323-bib-0063] Karakoc, D. B. , K. Barker , C. W. Zobel , and Y. Almoghathawi . 2020. “Social Vulnerability and Equity Perspectives on Interdependent Infrastructure Network Component Importance.” Sustainable Cities and Society 57: 102072.

[risa70323-bib-0064] Killick, R. , and I. Eckley . 2014a. “changepoint: An R package for Changepoint Analysis.” Journal of Statistical Software 58: 1–19.

[risa70323-bib-0065] Killick, R. , and I. A. Eckley . 2014b. “changepoint: An R Package for Changepoint Analysis.” Journal of Statistical Software 58: 1–19.

[risa70323-bib-0066] Koliou, M. , J. W. van de Lindt , T. P. McAllister , B. R. Ellingwood , M. Dillard , and H. Cutler . 2020. “State of the Research in Community Resilience: Progress and Challenges.” Sustainable and Resilient Infrastructure 5: 131–151. 10.1080/23789689.2017.1418547 PMC6508589 31080883

[risa70323-bib-0067] Kontou, E. , P. Murray‐Tuite , and K. Wernstedt . 2017. “Commuter Adaptation in Response to Hurricane Sandy's Damage.” Natural Hazards Review 18: 04016010.

[risa70323-bib-0068] Kullgren, J. T. , C. G. McLaughlin , N. Mitra , and K. Armstrong . 2012. “Nonfinancial Barriers and Access To Care for U.S. Adults.” Health Services Research 47: 462–485. 22092449 10.1111/j.1475-6773.2011.01308.x PMC3393009

[risa70323-bib-0069] Landsea, C. W. , and J. L. Franklin . 2013. “Atlantic Hurricane Database Uncertainty and Presentation of a New Database Format.” Monthly Weather Review 141: 3576–3592.

[risa70323-bib-0070] Lee, C.‐C. , M. Maron , and A. Mostafavi . 2022. “Community‐Scale Big Data Reveals Disparate Impacts of the Texas Winter Storm of 2021 and Its Managed Power Outage.” Humanities and Social Sciences Communications 9: 335. 36187845 10.1057/s41599-022-01353-8 PMC9510185

[risa70323-bib-0071] Levinson, D. , and H. Wu . 2020. “Towards a General Theory of Access.” Journal of Transport and Land Use 13: 129–158.

[risa70323-bib-0072] Logan, T. M. , M. J. Anderson , and A. C. Reilly . 2023. “Risk of Isolation Increases the Expected Burden From Sea‐Level Rise.” Nature Climate Change 13: 397–402

[risa70323-bib-0073] Logan, T. M. , M. J. Anderson , T. G. Williams , and L. Conrow . 2021. “Measuring Inequalities in Urban Systems: An Approach for Evaluating the Distribution of Amenities and Burdens.” Computers, Environment and Urban Systems 86: 101590.

[risa70323-bib-0074] Logan, T. M. , and S. D. Guikema . 2020. “Reframing Resilience: Equitable Access to Essential Services.” Risk Analysis 40: 1538–1553. 32402139 10.1111/risa.13492

[risa70323-bib-0075] Logan, T. M. , T. G. Williams , A. J. Nisbet , K. D. Liberman , C. T. Zuo , and S. D. Guikema . 2019. “Evaluating Urban Accessibility: Leveraging Open‐Source Data and Analytics to Overcome Existing Limitations.” Environment and Planning B: Urban Analytics and City Science 46: 897–913.

[risa70323-bib-0076] Long, E. F. , M. K. Chen , and R. Rohla . 2020. “Political Storms: Emergent Partisan Skepticism of Hurricane Risks.” Science Advances 6: eabb7906. 32917709 10.1126/sciadv.abb7906 PMC7486090

[risa70323-bib-0077] Loos, S. , D. Lallemant , F. Khan , et al. 2023. “A Data‐Driven Approach to Rapidly Estimate Recovery Potential to Go Beyond Building Damage After Disasters.” Communications Earth & Environment 4: 1–12.37325084

[risa70323-bib-0078] Mackrell, M. P. , K. J. Twilley , W. P. Kirk , L. Q. Lu , J. L. Underhill , and L. E. Barnes . 2013. “Discovering Anomalous Patterns in Network Traffic Data During Crisis Events.” In 2013 IEEE Systems and Information Engineering Design Symposium, 52–57. IEEE.

[risa70323-bib-0079] Maharjan, S. , N. Tilahun , and A. Ermagun . 2022. “Spatial Equity of Modal Access Gap to Multiple Destination Types Across Chicago.” Journal of Transport Geography 104: 103437.

[risa70323-bib-0080] Morgan, M. , M. Young , R. Lovelace , and L. Hama . 2019. “OpenTripPlanner for R.” Journal of Open Source Software 4: 1926.

[risa70323-bib-0081] Martens, K. 2012. “Justice in Transport as Justice in Accessibility: Applying Walzer's ‘Spheres of Justice’ to the Transport Sector.” Transportation 39: 1035–1053.

[risa70323-bib-0082] Marzuoli, A. , and F. Liu . 2018. “A Data‐Driven Impact Evaluation of Hurricane Harvey From Mobile Phone Data.” In 2018 IEEE International Conference on Big Data (Big Data), 3442–3451. IEEE.

[risa70323-bib-0083] Marzuoli, A. , and F. Liu . 2019. “Monitoring of Natural Disasters Through Anomaly Detection on Mobile Phone Data.” In 2019 IEEE International Conference on Big Data (Big Data), 4089–4098. IEEE.

[risa70323-bib-0084] McAllister, T. 2016. “Research Needs for Developing a Risk‐Informed Methodology for Community Resilience.” Journal of Structural Engineering 142: C4015008.

[risa70323-bib-0085] Meerow, S. , J. P. Newell , and M. Stults . 2016. “Defining Urban Resilience: A Review.” Landscape and Urban Planning 147: 38–49.

[risa70323-bib-0086] Mitsova, D. , A.‐M. Esnard , A. Sapat , and B. S. Lai . 2018. “Socioeconomic Vulnerability and Electric Power Restoration Timelines in Florida: The Case of Hurricane Irma.” Natural Hazards 94: 689–709.

[risa70323-bib-0087] Mitsova, D. , A. M. Esnard , A. Sapat , A. Lamadrid , M. Escaleras , and C. Velarde‐Perez . 2021. “Effects of Infrastructure Service Disruptions Following Hurricane Irma: Multilevel Analysis of Postdisaster Recovery Outcomes.” Natural Hazards Review 22: 04020055.

[risa70323-bib-0088] Mooney, P. , A. Y. Grinberger , M. Minghini , S. Coetzee , L. Juhasz , and G. Yeboah . 2021. “OpenStreetMap Data Use Cases During the Early Months of the COVID‐19 Pandemic.” In COVID‐19 Pandemic, Geospatial Information, and Community Resilience, 171–186. CRC Press.

[risa70323-bib-0089] Murphy, C. , and P. Gardoni . 2006. “The Role of Society in Engineering Risk Analysis: A Capabilities‐Based Approach.” Risk Analysis 26: 1073–1083. 16948698 10.1111/j.1539-6924.2006.00801.x

[risa70323-bib-0090] Nederhoff, K. , A. Giardino , M. van Ormondt , and D. Vatvani . 2019. “Estimates of Tropical Cyclone Geometry Parameters Based on Best‐Track Data.” Natural Hazards and Earth System Sciences 19: 2359–2370.

[risa70323-bib-0091] Newspapers, T. C. n.d. “Map: Hurricane Irma Power Outages.” https://data.tcpalm.com/storm‐power‐outages/. Accessed October 1, 2023.

[risa70323-bib-0092] NOAA National Centers for Environmental Information (NCEI) . 2022. “Digital Elevation Models Global Mosaic (Elevation Values)—Overview”.

[risa70323-bib-0005] OpenMobilityData . 2023. https://transitfeeds.com/. Accessed October 6, 2023.

[risa70323-bib-0094] O'Hare, P. , I. White , and A. Connelly . 2016. “Insurance as Maladaptation: Resilience and the ‘Business As Usual’ Paradox.” Environment and Planning C: Government and Policy 34: 1175–1193.

[risa70323-bib-0095] Pappalardo, L. , D. Pedreschi , Z. Smoreda , and F. Giannotti . 2015. “Using Big Data to Study the Link Between Human Mobility and Socio‐Economic Development.” In 2015 IEEE International Conference on Big Data (Big Data), 871–878. IEEE.

[risa70323-bib-0096] Park, J. , T. P. Seager , P. S. C. Rao , M. Convertino , and I. Linkov . 2013. “Integrating Risk and Resilience Approaches to Catastrophe Management in Engineering Systems.” Risk Analysis 33: 356–367. 22967095 10.1111/j.1539-6924.2012.01885.x

[risa70323-bib-0097] Penchansky, R. , and J. W. Thomas . 1981. “The Concept of Access: Definition and Relationship to Consumer Satisfaction.” Medical Care 19, no. 2: 127–140. 7206846 10.1097/00005650-198102000-00001

[risa70323-bib-0098] Podesta, C. , N. Coleman , A. Esmalian , F. Yuan , and A. Mostafavi . 2021. “Quantifying Community Resilience Based on Fluctuations in Visits to Points‐of‐Interest Derived From Digital Trace Data.” Journal of The Royal Society Interface 18: 20210158. 33906388 10.1098/rsif.2021.0158 PMC8086905

[risa70323-bib-0099] Rachunok, B. , and R. Nateghi . 2021. “Overemphasis on Recovery Inhibits Community Transformation and Creates Resilience Traps.” Nature Communications 12: 7331. 10.1038/s41467-021-27359-5 PMC8683504 34921147

[risa70323-bib-0100] Rufat, S. , E. Tate , C. T. Emrich , and F. Antolini . 2019. “How Valid Are Social Vulnerability Models?” Annals of the American Association of Geographers 109: 1131–1153. 39624068 10.1080/24694452.2018.1535887 PMC11611389

[risa70323-bib-0101] Sanders, B. F. , J. E. Schubert , D. T. Kahl , et al. 2023. “Large and Inequitable Flood Risks in Los Angeles, California.” Nature Sustainability 6: 47–57.

[risa70323-bib-0102] Saurman, E. 2016. “Improving Access: Modifying Penchansky and Thomas's Theory of Access.” Journal of Health Services Research & Policy 21: 36–39. 26377728 10.1177/1355819615600001

[risa70323-bib-0103] Shao, Y. , and W. Luo . 2026. “Realized Spatial Accessibility vs. Potential Spatial Accessibility in the United States: A Case Study Based on Geospatial Big Data.” Computers, Environment and Urban Systems 125: 102382.

[risa70323-bib-0104] Singh, S. R. , M. R. Eghdami , and S. Singh . 2014. “The Concept of Social Vulnerability: A Review From Disasters Perspectives.” International Journal of Interdisciplinary and Multidisciplinary Studies 1: 71–82.

[risa70323-bib-0105] Sovacool, B. K. 2017. “Don't Let Disaster Recovery Perpetuate Injustice.” Nature 549: 433. 28959978 10.1038/549433a

[risa70323-bib-0106] Spielman, S. E. , J. Tuccillo , D. C. Folch , et al. 2020. “Evaluating Social Vulnerability Indicators: Criteria and Their Application to the Social Vulnerability Index.” Natural Hazards 100: 417–436.

[risa70323-bib-0107] Svirsko, A. C. , T. Logan , C. Domanowski , and D. Skipper . 2022. “Developing Robust Facility Reopening Processes Following Natural Disasters.” Operations Research Forum 3: 39.

[risa70323-bib-0108] Swanson, T. , and S. Guikema . 2023. “Using Mobile Phone Data to Evaluate Access to Essential Services Following Natural Hazards.” Risk Analysis 44: 883–906. 37515569 10.1111/risa.14201

[risa70323-bib-0109] Swanson, T. , and S. Guikema . 2026. “Using Mobile Phone Data for Quantifying Large‐Scale Household‐Level Disaster Recovery.” Computers, Environment and Urban Systems 125: 102395.

[risa70323-bib-0110] Tariverdi, M. , M. Nunez‐Del‐Prado , N. Leonova , and J. Rentschler . 2023. “Measuring Accessibility to Public Services and Infrastructure Criticality for Disasters Risk Management.” Scientific Reports 13: 1569. 36709371 10.1038/s41598-023-28460-z PMC9884248

[risa70323-bib-0111] Tate, E. 2012. “Social Vulnerability Indices: A Comparative Assessment Using Uncertainty and Sensitivity Analysis.” Natural Hazards 63: 325–347.

[risa70323-bib-0006] Transitland . 2023. https://www.transit.land/terms. Last accessed October 6, 2023

[risa70323-bib-0112] Toole, J. L. , S. Colak , B. Sturt , L. P. Alexander , A. Evsukoff , and M. C. González . 2015. “The Path Most Traveled: Travel Demand Estimation Using Big Data Resources.” Transportation Research Part C: Emerging Technologies 58: 162–177.

[risa70323-bib-0113] Turner, B. L. , R. E. Kasperson , P. A. Matson , et al. 2003. “A Framework for Vulnerability Analysis in Sustainability Science.” Proceedings of the National Academy of Sciences 100: 8074–8079. 10.1073/pnas.1231335100 PMC166184 12792023

[risa70323-bib-0114] United Nations Educational . Scientific and Cultural Organization . and World Bank . 2018. Culture in City Reconstruction and Recovery. UNESCO. © UNESCO and World Bank.

[risa70323-bib-0115] United Nations Office for Disaster Response . 2022. “Global Assessment Report on Disaster Risk Reduction 2022: Our World at Risk: Transforming Governance for a Resilient Future”.

[risa70323-bib-0116] U.S. Census Bureau . 2017. “Types of Computers and Internet Subscriptions.” American Community Survey, 1‐Year Estimates, Table ID: S2801.

[risa70323-bib-0117] U.S. Census Bureau . 2021. “Florida 2017‐2021 American Community Survey Five‐Year Population Estimates.” American Community Survey, Five‐Year Estimates.

[risa70323-bib-0118] Van Ormondt, M. , K. Nederhoff , and A. van Dongeren . 2020. “Delft Dashboard: A Quick Set‐Up Tool for Hydrodynamic Models.” Journal of Hydroinformatics 22: 510–527.

[risa70323-bib-0119] Vargas‐Muñoz, J. E. , S. Lobry , A. X. Falcão , and D. Tuia . 2019. “Correcting Rural Building Annotations in OpenStreetMap Using Convolutional Neural Networks.” ISPRS Journal of Photogrammetry and Remote Sensing 147: 283–293.

[risa70323-bib-0120] Wang, P. , T. Hunter , A. M. Bayen , K. Schechtner , and M. C. González . 2012. “Understanding Road Usage Patterns in Urban Areas.” Scientific Reports 2: 1001. 23259045 10.1038/srep01001 PMC3526957

[risa70323-bib-0121] Wang, Q. , and J. E. Taylor . 2014. “Quantifying Human Mobility Perturbation and Resilience in Hurricane Sandy.” PLoS One 9: e112608. 25409009 10.1371/journal.pone.0112608 PMC4237337

[risa70323-bib-0122] Washington, V. , S. Guikema , J.‐L. Mondisa , and A. Misra . 2023. “A Data‐Driven Method for Identifying the Locations of Hurricane Evacuations from Mobile Phone Location Data.” Risk Analysis 44: 390–407. 37544906 10.1111/risa.14188

[risa70323-bib-0123] Widener, M. J. 2018. “Spatial Access to Food: Retiring the Food Desert Metaphor.” Physiology & Behavior 193: 257–260. 29454842 10.1016/j.physbeh.2018.02.032

[risa70323-bib-0124] Widener, M. J. , L. Minaker , S. Farber , et al. 2017. “How Do Changes in the Daily Food and Transportation Environments Affect Grocery Store Accessibility?” Applied Geography 83: 46–62.

[risa70323-bib-0125] Williams, T. G. , T. M. Logan , C. T. Zuo , K. D. Liberman , and S. D. Guikema . 2020. “Parks and Safety: A Comparative Study of Green Space Access and Inequity in Five US Cities.” Landscape and Urban Planning 201: 103841.

[risa70323-bib-0126] Wilson, R. , E. Zu Erbach‐Schoenberg , M. Albert , et al. 2016. “Rapid and Near Real‐Time Assessments of Population Displacement Using Mobile Phone Data Following Disasters: The 2015 Nepal Earthquake.” *PLOS Currents* 8. https://currents.plos.org/disasters/article/rapid‐and‐near‐real‐time‐assessments‐of‐population‐displacement‐using‐mobile‐phone‐data‐following‐disasters‐the‐2015‐nepal‐earthquake/. 10.1371/currents.dis.d073fbece328e4c39087bc086d694b5c PMC4779046 26981327

[risa70323-bib-0127] Yabe, T. , N. K. Jones , P. S. C. Rao , M. C. Gonzalez , and S. V. Ukkusuri . 2022. “Mobile Phone Location Data for Disasters: A Review From Natural Hazards and Epidemics.” Computers, Environment and Urban Systems 94: 101777.

[risa70323-bib-0128] Yabe, T. , Y. Sekimoto , K. Tsubouchi , and S. Ikemoto . 2019. “Cross‐comparative Analysis of Evacuation Behavior After Earthquakes Using Mobile Phone Data.” PLoS One 14: e0211375. 30785908 10.1371/journal.pone.0211375 PMC6382263

[risa70323-bib-0129] Yabe, T. , K. Tsubouchi , N. Fujiwara , Y. Sekimoto , and S. V. Ukkusuri . 2020. “Understanding Post‐Disaster Population Recovery Patterns.” Journal of the Royal Society Interface 17: 20190532. 32070218 10.1098/rsif.2019.0532 PMC7061695

[risa70323-bib-0130] Yabe, T. , S. V. Ukkusuri , and P. S. C. Rao . 2019. “Mobile Phone Data Reveals the Importance of Pre‐Disaster Inter‐City Social Ties for Recovery After Hurricane Maria.” Applied Network Science 4: 98.

[risa70323-bib-0131] Yin, L. , J. Chen , H. Zhang , et al. 2020. “Improving Emergency Evacuation Planning With Mobile Phone Location Data.” Environment and Planning B: Urban Analytics and City Science 47: 964–980.

[risa70323-bib-0132] Yu, M. , C. Yang , and Y. Li . 2018. “Big Data in Natural Disaster Management: A Review.” Geosciences Journal 8: 165.

